# Full-length transcript sequencing and comparative transcriptomic analysis to evaluate the contribution of osmotic and ionic stress components towards salinity tolerance in the roots of cultivated alfalfa (*Medicago sativa* L.)

**DOI:** 10.1186/s12870-019-1630-4

**Published:** 2019-01-21

**Authors:** Dong Luo, Qiang Zhou, Yuguo Wu, Xutian Chai, Wenxian Liu, Yanrong Wang, Qingchuan Yang, Zengyu Wang, Zhipeng Liu

**Affiliations:** 1 State Key Laboratory of Grassland Agro-ecosystems, Key Laboratory of Grassland Livestock Industry Innovation, Ministry of Agriculture and Rural Affairs, College of Pastoral Agriculture Science and Technology, Lanzhou University, Lanzhou, 730000 People’s Republic of China; 2 Institute of Animal Sciences, Chinese Academy of Agricultural Sciences, Beijing, 100000 People’s Republic of China; 3 Core Research & Transformation, Noble Research Institute, Ardmore, OK 73401 USA

**Keywords:** Alfalfa, Antioxidative defense, Differentially expressed genes, Full-length transcripts, Physiological shifts, Salinity stress

## Abstract

**Background:**

Alfalfa is the most extensively cultivated forage legume. Salinity is a major environmental factor that impacts on alfalfa’s productivity. However, little is known about the molecular mechanisms underlying alfalfa responses to salinity, especially the relative contribution of the two important components of osmotic and ionic stress.

**Results:**

In this study, we constructed the first full-length transcriptome database for alfalfa root tips under continuous NaCl and mannitol treatments for 1, 3, 6, 12, and 24 h (three biological replicates for each time points, including the control group) via PacBio Iso-Seq. This resulted in the identification of 52,787 full-length transcripts, with an average length of 2551 bp. Global transcriptional changes in the same 33 stressed samples were then analyzed via BGISEQ-500 RNA-Seq. Totals of 8861 NaCl-regulated and 8016 mannitol-regulated differentially expressed genes (DEGs) were identified. Metabolic analyses revealed that these DEGs overlapped or diverged in the cascades of molecular networks involved in signal perception, signal transduction, transcriptional regulation, and antioxidative defense. Notably, several well characterized signalling pathways, such as CDPK, MAPK, CIPK, and PYL-PP2C-SnRK2, were shown to be involved in osmotic stress, while the SOS core pathway was activated by ionic stress. Moreover, the physiological shifts of catalase and peroxidase activity, glutathione and proline content were in accordance with dynamic transcript profiles of the relevant genes, indicating that antioxidative defense system plays critical roles in response to salinity stress.

**Conclusions:**

Overall, our study provides evidence that the response to salinity stress in alfalfa includes both osmotic and ionic components. The key osmotic and ionic stress-related genes are candidates for future studies as potential targets to improve resistance to salinity stress via genetic engineering.

**Electronic supplementary material:**

The online version of this article (10.1186/s12870-019-1630-4) contains supplementary material, which is available to authorized users.

## Background

Plants undergo constant exposure to highly variable environmental stresses during their life cycles, with salinity stress representing the leading constraint to growth and productivity, which is responsible for quality and yield [1]. In general, salinity interferes with plant growth because it imposes two main stresses on plants: hyperosmotic pressure, resulting from the low water availability, and ion toxicity (mainly Na^+^), arising from solute imbalances [2]. For plants to survive under this stress condition, they will employ intricate defense mechanisms through a series of drastic physiological and biochemical changes [3]. These modifications include maintaining the integrity of the cell membrane, regulating water balance, scavenging reactive oxygen species (ROS), and accumulating compatible solutes, as well as reinstating cellular ionic equilibrium [4], which are all dedicated to reducing the osmotic or ionic damage caused by salinity.

The physiological responses of plants acclimating to unfavourable environments are all initiated upon the activation of cascades of molecular networks within the signalling pathways [5]. In the signalling pathways, high salinity level often triggers an increase in cytosolic Ca^2+^, ROS, and ABA, which are critical signal transduction components [6]. Activated Ca^2+^, ROS, and ABA signalling cascades further alter plant transcriptomes by regulating downstream transcription factors (TFs), such as AP2-EREBPs, MYBs, and bHLHs. Thereafter, these TFs can cause changes in the expression of various osmotic stress-responsive genes, such as *P5CSs* and *COR15As*, and ionic stress-responsive genes, such as *NHXs* and *HKTs*, which ultimately contribute to plant salinity tolerance [7, 8]. Recently, several well characterized signalling pathways of plants responding to salinity stress have been revealed, such as the calcium-dependent protein kinase (CDPK) pathway, which plays a critical role in osmotic stress response [9]; the salt overly sensitive (SOS) pathway, which is activated by Ca^2+^ spikes from the cytoplasm and overcomes ionic damage by maintaining cellular ion homeostasis [10]; and the calcineurin B-like proteins–CBL-interacting protein kinases (CBL-CIPK) module, which is essential to combating both osmotic and ionic stress [11]. Despite the progress that has been made in detailing these processes, the underlying mechanisms of plants’ response to salinity need further exploration, especially in non-model plants.

Alfalfa (*Medicago sativa* L.), is the most widely cultivated perennial forage legume, and more than 40 million hectares are planted worldwide [12]. This species is referred to as the “queen of forages” and is used as hay, silage, and pasture for ruminants and dairy production [13]. Moreover, alfalfa possesses considerable potential as a biofuel feedstock for ethanol production [14]. In China, alfalfa plantation areas are mainly distributed in the northern, northwestern, and northeastern regions [15]. Unfortunately, soil salinization is dramatically increasing in those areas, which dramatically limits the productivity and persistence of alfalfa [16]. Therefore, it is imperative to perform studies on the molecular mechanisms of adaptation to salinity stress in alfalfa.

Previous studies have shown that overexpression of the stress-associated genes encoding the compatible solute *AgcodA* [17], ion transporter *SeNHX1* [18], protein kinase *AtNDPK2* [19], or TFs *GmDREB1* [20] and *GsWRKY20* [21] results in enhanced tolerance to salinity stress in alfalfa. Given the relatively low-throughput characteristics of genetic-based approaches, large-scale potential genes involved in alfalfa responses to adverse salinity stimuli have been studied via next-generation sequencing (NGS) technologies. Postnikova et al. (2013) performed the transcriptional profiling of alfalfa whole roots under NaCl stress for 7 days in two distinct salinity-tolerant germplasms; their results showed that salinity-responsive genes are mainly involved in stabilization of the plasma membrane and several salinity-responsive TF families [22]. Lei et al. (2018) comparatively analyzed the leaf transcriptomes under NaCl stress for 7 days between two different salinity-tolerant alfalfa cultivars, which revealed that plant hormone interactions is a vital regulator in alfalfa to maintain specific physiological status for adaptation to salinity stress [23]. Furthermore, a de novo transcriptional analysis of whole alfalfa seedlings treated with saline–alkaline solutions for 0, 1, and 7 days indicated that antioxidant capacity was one of the central mechanisms underlying alfalfa’s saline–alkaline stress tolerance [24]. However, these studies mainly focused on genotype-specific salinity tolerance mechanisms or more complex saline–alkaline tolerance mechanisms, systematic consensus on the comparative damage caused by osmotic versus ionic stresses when alfalfa is subjected to salinity is still lacking. And also, even with these transcriptional-based NGS methods, the disadvantage was clear, such as the short lengths of sequencing reads, which greatly hinders its ability to estimate transcript abundance at genome-wide scale.

Luckily, the PacBio RSII third-generation sequencing technology can overcome these limitations. Compared with traditional NGS technologies, this technology accomplishes single molecule real-time (SMRT) isoform sequencing (Iso-Seq) with long read lengths, uniform coverage, and high accuracy, which renders PacBio RSII very effective at capturing the full catalogue of transcripts and constructs of a comprehensive transcriptome for species without genome sequence [25]. Furthermore, RNA-Seq based on the BGISEQ-500 platform has been applied to gene expression comparisons of different species, developmental stages, and stresses [26, 27]. Currently, to our knowledge, genome-wide transcriptomic analysis of the salinity-responsive genes has not been reported in alfalfa root tips, where is the primary site for the perception of hyperosmotic pressure and ion toxicity [28, 29]. Thus, for the first time, we applied the Iso-Seq protocol to generate a full-length reference transcriptome for alfalfa root tips during continuous NaCl (an iso-osmotic stressor) and mannitol (a non-ionic osmotic stressor) treatments and then performed a gene expression comparison for the same stressed samples at the transcriptional scale using BGISEQ-500 RNA-Seq. Moreover, the physiological effects of NaCl and mannitol treatments on ROS accumulation and cell damage, as well as underlying antioxidant and osmoprotectant responses, were determined. The results of this study will help us to understand the contribution of the two osmotic and ionic components towards salinity tolerance in alfalfa.

## Results

### Physiology assay

Since high concentrations of NaCl and mannitol cause severe inhibition of growth, moderate concentrations, namely 250 mM NaCl and 400 mM mannitol, were used for physiological and transcriptome analyses (Additional file 1: Figure S1).

**Fig. 1:**
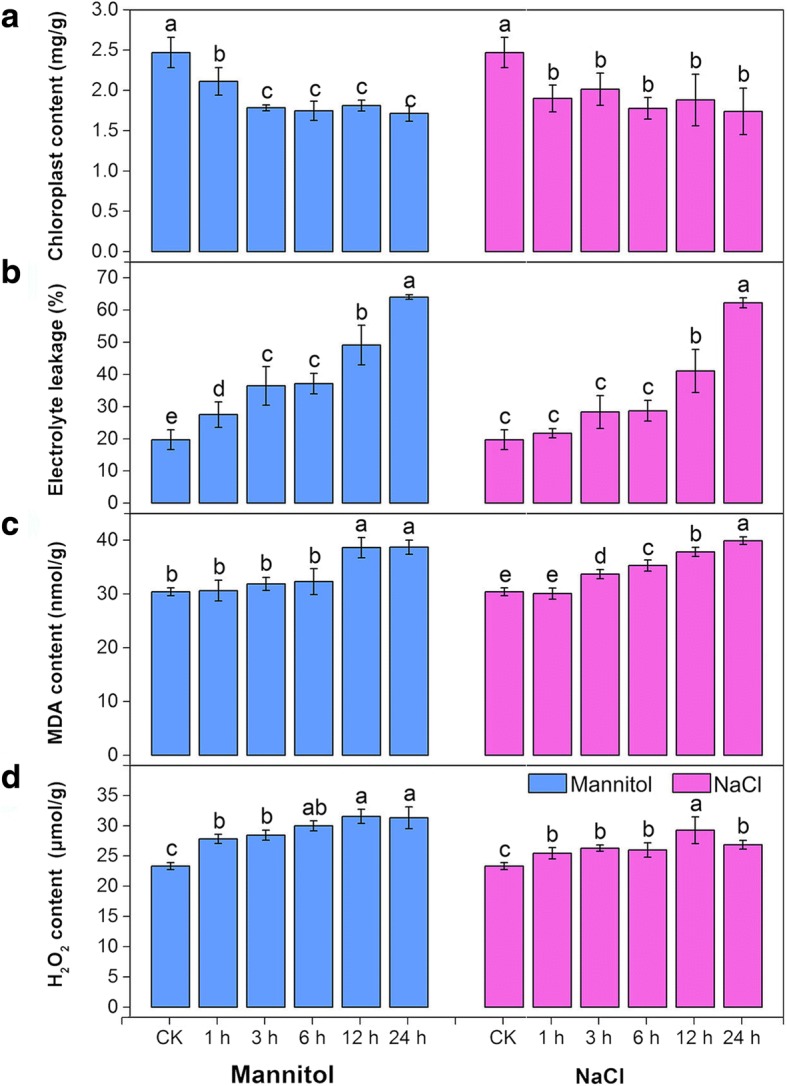
Analyses of dynamic physiological effects under continuous NaCl and mannitol stress. **a** Chlorophyll content. **b** Electrolyte leakage. **c** MDA content. **d** H_2_O_2_ content. The results are the means and SDs of three replicates. Different letters above the bars indicate significant difference at the 0.05 level according to Duncan’s multiple range test

When alfalfa seedlings were cultivated under abiotic stress, severe symptoms of injury appeared. As shown in Fig. 1a, chloroplast content decreased to 29.50 and 30.56% of the control content at 24 h under NaCl and mannitol stress, respectively. Both NaCl and mannitol stresses caused significant increases in ion leakage in alfalfa seedlings, and the changes became more evident with increasing time (Fig. 1b). In contrast, both stresses caused a gradual slight increase in malonaldehyde (MDA) content (Fig. 1c).

Since oxidative injury is generated under abiotic stress by the formation of ROS in plants, H_2_O_2_ contents in alfalfa seedlings were measured. Under NaCl stress, the content of H_2_O_2_ increased moderately, peaking at 12 h and declining slightly at 24 h. In contrast, under mannitol stress, the content of H_2_O_2_ increased greatly and remained stable after 12 h (Fig. 1d).

**Fig. 2:**
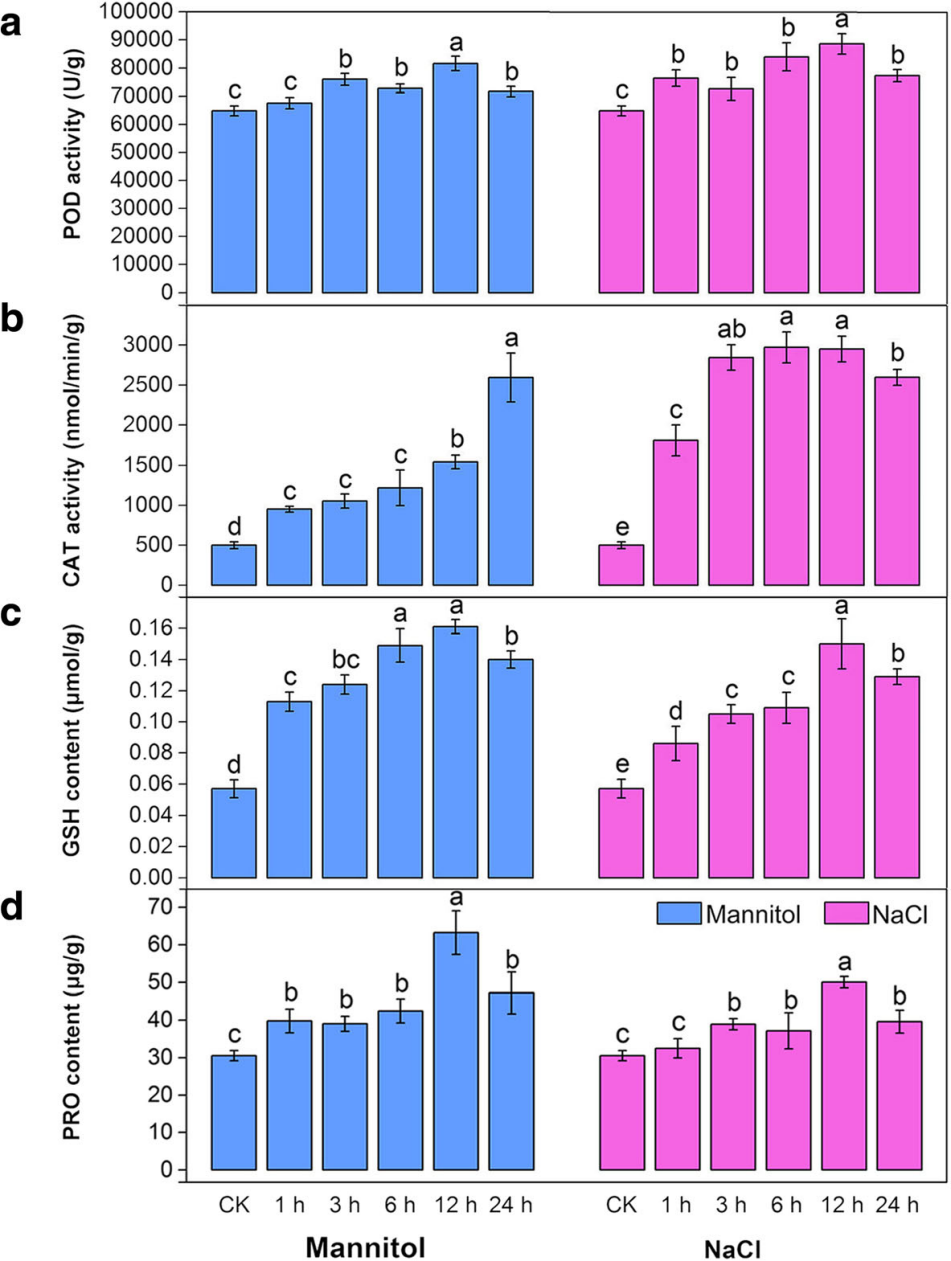
Analyses of dynamic physiological responses under continuous NaCl and mannitol stress. **a** POD activity. **b** CAT activity. **c** GSH content. **d** PRO content. The results are the means and SDs of three replicates. Different letters above the bars indicate significant difference at the 0.05 level according to Duncan’s multiple range test

To evaluate whether the cellular antioxidant defence system was activated, the activities of key antioxidant enzymes such as peroxidase (POD) and catalase (CAT) and the contents of antioxidants such as reduced glutathione (GSH) were tested. As indicated in Fig. 2a, both NaCl and mannitol stresses induced a significant increase in POD activity compared with the control treatments. In parallel with the POD activity, under NaCl stress, the activities of CAT increased to higher levels than those in the control groups from 1 to 3 h; the activities then maintained steady levels from 3 to 12 h but showed a slight decrease at 24 h. Under mannitol stress, the activities of CAT showed a significant increase from 1 to 24 h (Fig. 2b). In addition, the GSH content showed similar patterns under both stresses, it increased significantly from 0 to 6 h and then peaked at 12 h (Fig. 2c).

Because proline (PRO) acts as an important osmoprotectant in plants and provides abiotic stress tolerance, the content of PRO was also examined. Figure 2d shows that both NaCl and mannitol stresses induced a pronounced increase in the contents of PRO compared with the control treatments and peaked at 12 h. NaCl stress induced a lower increase in the contents of PRO, which increased by 63.94% compared to the mannitol stress (107.11%) at 12 h.

### Iso-Seq, assembly, annotation, and CDSs prediction

The pooled total RNA for all 33 samples receiving one (CK, without treatment) control, (S1, S2, S3, S4, and S5, which represent 1, 3, 6, 12, and 24 h after NaCl tretment, respectively) NaCl, and five (M1, M2, M3, M4, and M5, which represent 1, 3, 6, 12, and 24 h after mannitol treatment, respectively) mannitol at different time points was used to generate an informative reference transcriptome database; every time point involved three biological replicates. Four Iso-Seq libraries were constructed, and 12 cells in total were sequenced on a PacBio RS II platform; a total of 1,315,535,395 raw data (448,454 reads) were ultimately generated (Table 1). After removing the redundancy and merging the high-quality consensus transcripts of each library, a total of 52,787 full-length transcripts was obtained; 26,600, 20,230, 5389, and 3596 transcripts were identified in the 1–2 kb, 2–3 kb, 3–6 kb, and 5–10 kb libraries, respectively (Table 1). The length of these 52,787 full-length transcripts ranged from 303 to 8445 bp; the mean size was 2551 bp, and the N50 length was 2928 bp. There were 34,193 (64.78%) transcripts that were longer than 2000 bp. Additional file 1: Figure S2a shows the quality of the assembly transcripts in more detail. All of the sequencing reads were deposited into the NCBI Sequence Read Archive (SRA) database (SRR7091350–53).

**Table 1:** Summary of the full-length transcript sequence data analysis

Library	Cell Number	Reads of Insert	Read Bases of Insert (bp)	Mean Read Length of Insert (bp)	Mean Read Quality of Insert	Mean Number of Passes	Transcripts
1–2 kb	4	187,278	431,482,422	2303	0.92	9	26,600
2–3 kb	4	148,823	405,714,966	2726	0.91	7	20,230
3–6 kb	2	51,290	189,968,606	3703	0.88	4	5389
5–10 kb	2	61,063	288,369,401	4722	0.87	4	3596
Total	12						52,787

BLASTx (*E*-value ≤10^− 5^) searches were carried out to perform functional annotations with transcripts against seven public databases (Additional file 1: Figure S2b; Additional file 2: Table S1), including the National Center for Biotechnology Information (NCBI) non-redundant protein sequence (Nr), NCBI non-redundant nucleotide sequence (Nt), SwissProt, InterPro, Clusters of Orthologous Groups of proteins (COG), Gene Ontology (GO), and Kyoto Encyclopedia of Genes and Genomes (KEGG) databases. The number of transcripts annotated in these seven databases ranged from 15,661 (29.67%, GO) to 51,980 (98.47%, Nr), and 52,687 (99.81%) and 9006 (17.06%) transcripts were annotated in at least one database and in all databases, respectively.

Based on the four public protein databases in the priority order of NR, SwissProt, KEGG, and COG, the CDSs of 51,895 (98.31%) transcripts were predicted by BLASTx (*E*-value ≤10^− 5^), whereas CDS predictions of 69 transcripts (0.13%) were made via ESTScan software; the mean lengths were 854 bp and 1737 bp, respectively (Table 2). Of these CDSs, 387 (0.73%) were longer than 3000 bp, 39,766 (75.33%) ranged from 500 bp to 3000 bp, and 11,811 (22.38%) were shorter than 500 bp (Additional file 1: Figure S2c). Moreover, among the 52,787 full-length transcripts, nearly one-third (15,661) were assigned to 2146 GO annotations distributed across 49 sub-functional groups, with 42.61% in the biological process, 40.46% in the molecular function, and 16.93% in the cellular component groups (Additional file 1: Figure S2d). Additionally, all of the transcripts were further assigned to the KEGG pathway databases. A total of 42,369 full-length transcripts were annotated in 135 individual KEGG pathways (Additional file 1: Figure S2e).

**Table 2:** The quality of predicted CDSs from full-length transcripts

Software	Total Number	Total Length	Mean Length	N50	N70	N90	GC(%)
Blast	51,895	44,348,718	854	1119	789	462	42
ESTScan	69	119,898	1737	2091	1827	813	48
Overall	51,964	44,468,616	855	1119	792	462	42

### BGISEQ-500 RNA-Seq

Thirty-three cDNA libraries (three libraries for each of the 11 time points) were designed for high-throughput RNA-Seq. In total, 769,932,394 raw reads were generated by the BGISEQ-500 platform, yielding a total of 740,197,889 high-quality clean reads with an average of 22,430,239 clean reads for each library. All of the sequence read data were deposited in the NCBI SRA database (SRR7160314–15, 22–23, 25–49, 51–52, 56–57). To identify the genes corresponding to these clean reads in each library, the clean reads were then mapped to the reference genes of the “MSA” alfalfa full-length transcriptome database via Bowtie2 software. An average of 70.85% of clean reads uniquely mapped to the alfalfa full-length transcripts. The RSEM software package was used to evaluate relative abundance values by calculating the fragments per kilobase per million fragments mapped (FPKM). Finally, a total of 51,896 transcripts were identified in all 33 samples, with more than 40,000 genes from each library. Additional details of the quality of the sequencing data are shown in Additional file 1: Figure S3 and Additional file 2: Table S2.

### Verification of gene expression

**Fig. 3:**
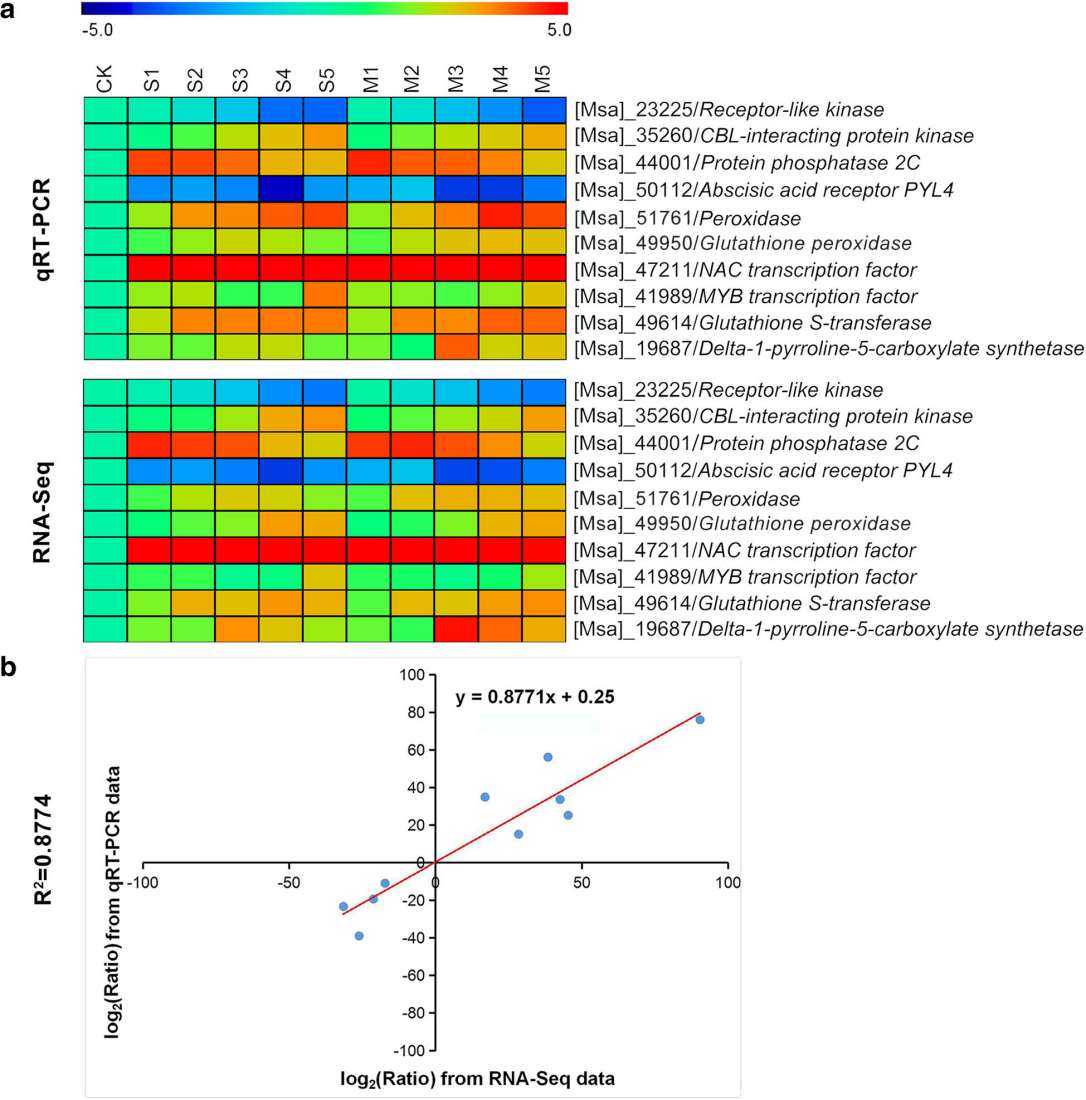
The expression pattern of ten selected genes identified by RNA-Seq was verified by qRT-PCR. **a** Heat map showing the expression changes (log_2_-fold change) in response to the CK to M5 treatments for each candidate gene as measured by RNA-Seq and qRT-PCR. **b** Scatter plot showing the changes in the expression (log_2_-fold change) of selected genes based on RNA-Seq via qRT-PCR. The gene expression levels are indicated by coloured bars

To confirm the reliability of our transcriptome data, the expression fold changes of 10 candidate transcripts were determined via quantitative real-time PCR (qRT-PCR) and further compared with those revealed by the RNA-Seq data. In our analysis, a positive correlation coefficient (R^2^ = 0.8774) was obtained by linear regression analysis, suggesting that the expression of these selected genes in our transcriptome data was generally in good agreement with the qRT-PCR results (Fig. 3).

### Differentially expressed genes (DEGs) analysis

**Fig. 4:**
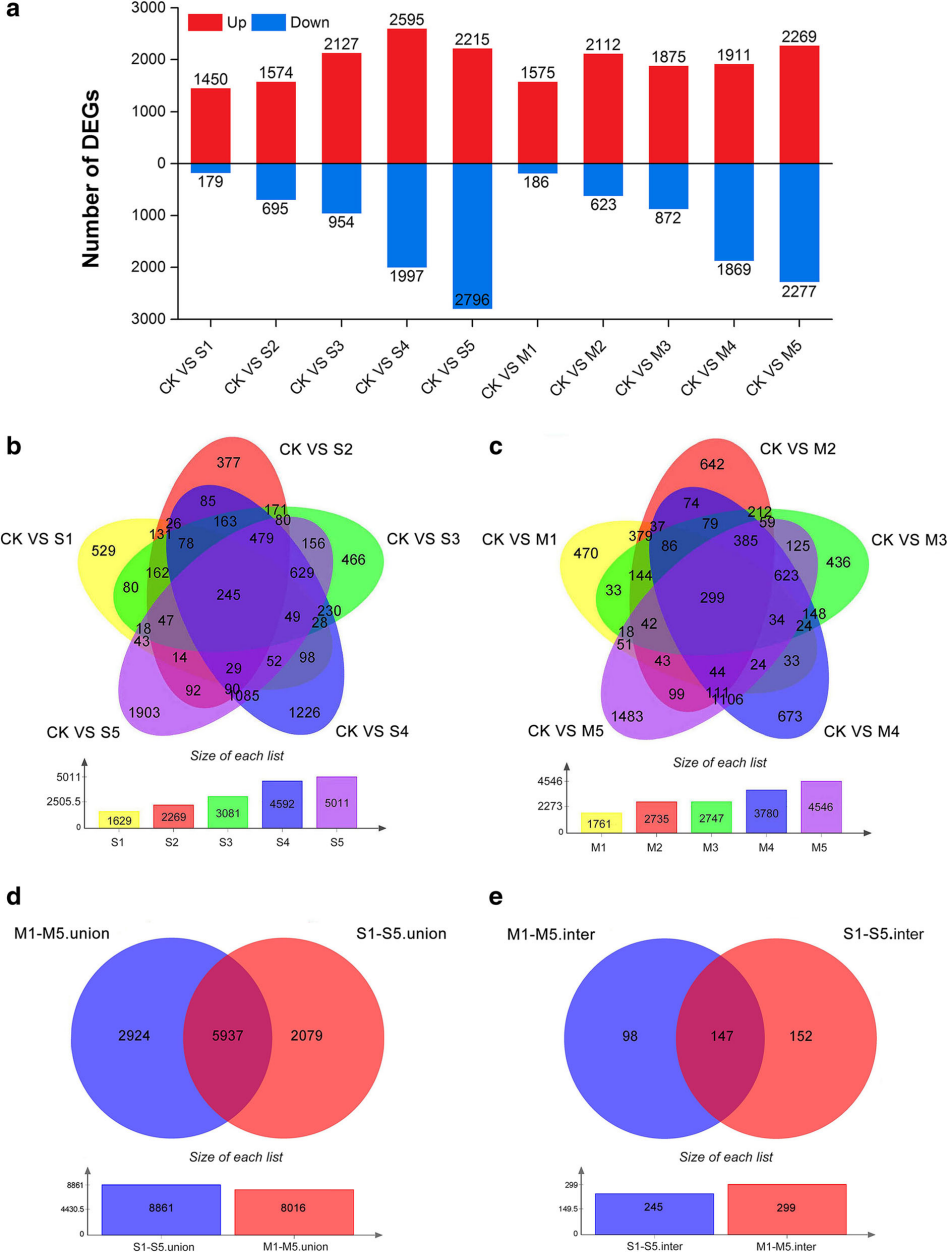
Summary of the differentially expressed genes. **a** A summary of the numbers of up- and down-regulated DEGs. **b** The numbers of DEGs expressed at one NaCl-treated time point and at overlapping time points compared with the control. **c** The numbers of DEGs expressed at one mannitol-treated time point and at overlapping time points compared with the control. **d** The combined set of all analyzed time points of NaCl- and mannitol-regulated DEGs at each treatment and those overlapping between the two treatments. **e** The intersecting set of all analyzed time points of NaCl- and mannitol-regulated DEGs at each treatment and those overlapping between the two treatments

Upon comparison with the control group, the genes that met the default criteria with an absolute value of fold change ≥4 and a divergence probability ≥0.8 found by NOISeq software were assigned as DEGs (Fig. 4a). In the S1, S2, S3, S4, and S5 groups, 1629, 2269, 3081, 4592, and 5011 DEGs in response to 250 mM NaCl were detected, respectively (Fig. 4b). In the M1, M2, M3, M4, and M5 groups, 1761, 2735, 2747, 3780, and 4546 DEGs in response to 400 mM mannitol were detected, respectively (Fig. 4c). Additional file 1: Figure S4 shows an overview of the continuous dynamic changes in DEG expression levels associated with NaCl and mannitol stress.

Of the 8861 NaCl-regulated and 8016 mannitol-regulated DEGs with significant expression changes at one or more time points, 5937 DEGs were co-regulated by both NaCl and mannitol stresses, accounting for 67.00 and 74.06% of the total number of DEGs, respectively (Fig. 4d). Of these co-regulated DEGs, 147 exhibited significant expression changes at all NaCl and mannitol treatment time points, 143 were up-regulated and 4 were down-regulated (Fig. 4e; Additional file 2: Table S3). Furthermore, 2924 and 2079 DEGs were exclusively expressed during at least one time point in response to NaCl and mannitol, respectively (Fig. 4d). Of these specifically regulated DEGs, 98 (85 up-regulated and 13 down-regulated) and 152 (96 up-regulated, 54 down-regulated, and 2 dynamically regulated) exhibited significant changes in expression across all the time points under NaCl and mannitol stress, respectively (Fig. 4e; Additional file 2: Table S3).

### Identification of TFs

Of the 8861 NaCl-regulated and 8016 mannitol-regulated DEGs, 162 were found to belong to 30 TF families for NaCl, and 138 were found to belong to 28 TF families for mannitol (Additional file 2: Table S4). Totals of 26 TF families were common to these two stress conditions. Members of the GRAS (28 and 26 for NaCl and mannitol, respectively) family were the most abundant, followed by the MYB (21 and 15), AP2-EREBP (13 and 15), and NAC (12 and 12) families. In addition to the abovementioned TFs, we also identified four TF families—ARR-Bs (3), bZIPs (2), C2C2-CO-likes (1), and LOB (1)—that were induced only by NaCl stress, and two TF families—CPPs (1) and E2F-DP (1) —that were specifically induced by mannitol stress.

**Fig. 5:**
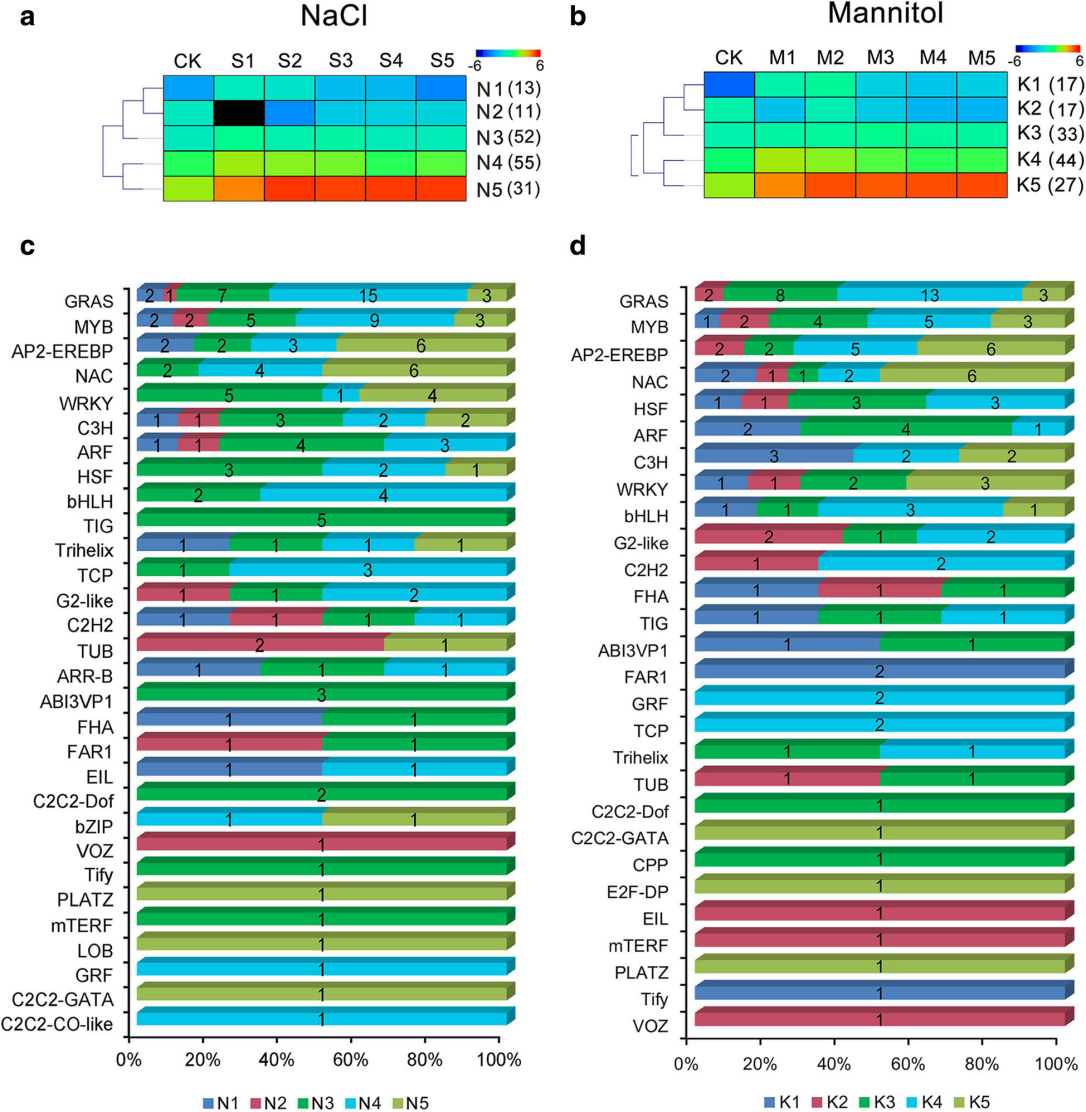
Dynamics of transcription factor accumulation profiles. **a** and **b** showing the dendrogram of the TFs; 162 and 138 significantly differentially expressed TFs from each time point of NaCl and mannitol stress clustered into five lineages respectively (N1–5 in NaCl stress and K1–5 in mannitol stress) using MEV 4.9 software. **c** and **d** show the distribution of TF families among N1–5 and K1–5, respectively

Furthermore, based on Self-Organizing Tree Algorithm in the MEV 4.9 software, the expression patterns of differentially expressed TFs were clustered into five groups for each of the two stresses (N1–N5 for NaCl stress and K1–K5 for mannitol stress) (Fig. 5). Our data showed that most of the TF families have a similar expression pattern under NaCl and mannitol stress, such as GRAS, AP2-EREBP, MYB, NAC, and G2-like, while a few TF families showed different expression pattern between the two stresses, such as C2H2, FAR, TIG, and Trihelix.

### GO functional analysis

**Fig. 6:**
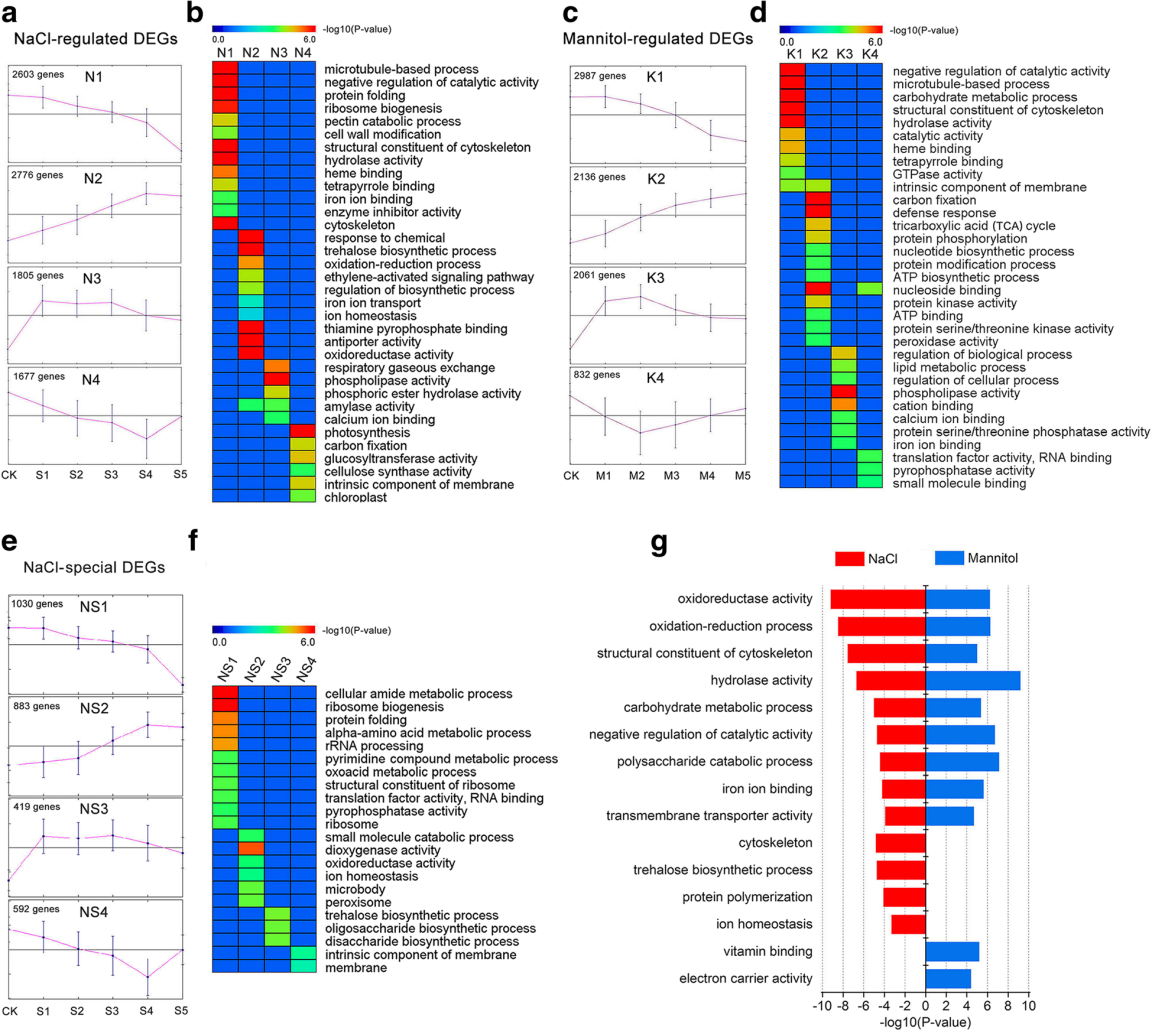
Dynamic progression of alfalfa transcriptome under NaCl and mannitol stress. **a**, **c**, and **e** showing the dynamic expression of NaCl-regulated DEGs (N1–4), mannitol-regulated DEGs (K1–4), and NaCl-specific DEGs (NS1–4), respectively, by K-means clustering. **b**, **d**, and **f** showing the functional enrichment among the clusters. **g** Functional enrichment among the DEGs shared between NaCl and mannitol stress. The names of the GO categories are listed along the y-axis. The degree of GO enrichment is represented by the -log_10_ (*P*-value)

To determine whether the accumulated transcripts were functionally involved in stress response/defence processes, we applied GO category enrichment analysis to speculate the function of the DEGs activated under NaCl and mannitol stress. Using K-Mean clustering algorithm in MEV 4.9 software, all 8861 NaCl-regulated and 8016 mannitol-regulated DEGs were clustered into four main groups (designated N1–4 and K1–4, respectively) with highly similar temporal expression patterns (Fig. 6a, c). The groups of genes in each cluster showed confident enrichments for particular functional categories (corrected *P* < 0.05). In general, similar functional categories were enriched between NaCl and mannitol stress (Fig. 6b, d). Under both stress conditions, continuously down-regulated genes (N1 and K1) were suggested to be required for “negative regulation of catalytic activity”, “microtubule-based process”, “structural constituent of cytoskeleton”, “hydrolase activity”, “heme binding”, and “tetrapyrrole binding”, whereas genes whose expression peaked during the early stressed stages (N3 and K3) were predicted to be involved in “phospholipase activity” and “calcium ion binding” were greatly enriched. However, the functional categories of stress-responsive genes were also observed to be different between the two-stressed alfalfa plants. Under NaCl stress, continuously up-regulated genes (N2) were mainly involved in “ion homeostasis”, “antiporter activity”, “trehalose biosynthetic process”, “thiamine pyrophosphate binding”, and “ethylene-activated signalling pathway”, while the continuously up-regulated genes (K2) during mannitol stress were “carbon fixation”, “tricarboxylic acid cycle”, “protein phosphorylation”, “nucleotide biosynthetic process”, and “ATP binding”. To further separate the ionic stress-related processes, 2924 NaCl-specific DEGs, clustered into four main groups (NS1–4), were used for GO category enrichment analysis (Fig. 6e, f). As expected, “cellular amide metabolic process”, “ion homeostasis”, “trehalose biosynthetic process”, and “small molecule catabolic process” were enriched in the NaCl-specific genes.

Furthermore, we performed a comparative analysis of GO category enrichment between DEGs under NaCl and mannitol stress. Totals of 13 and 11 GO terms were identified as over-representations based on an FDR < 0.05, respectively (Fig. 6g). Notably, the DEGs of both stresses were commonly involved in nine GO categories: “oxidoreductase activity”, “oxidation-reduction process”, “structural constituent of cytoskeleton”, “hydrolase activity”, “carbohydrate metabolic process”, “negative regulation of catalytic activity”, “polysaccharide catabolic process”, “iron ion binding”, and “transmembrane transporter activity”. DEGs belonging to the four GO categories of “cytoskeleton”, “trehalose biosynthetic process”, “protein polymerization”, and “ion homeostasis” contained only the DEGs from NaCl stress, while the two GO categories “vitamin binding” and “electron carrier activity” were exclusively present under mannitol stress.

### KEGG pathway enrichment analysis

**Fig. 7:**
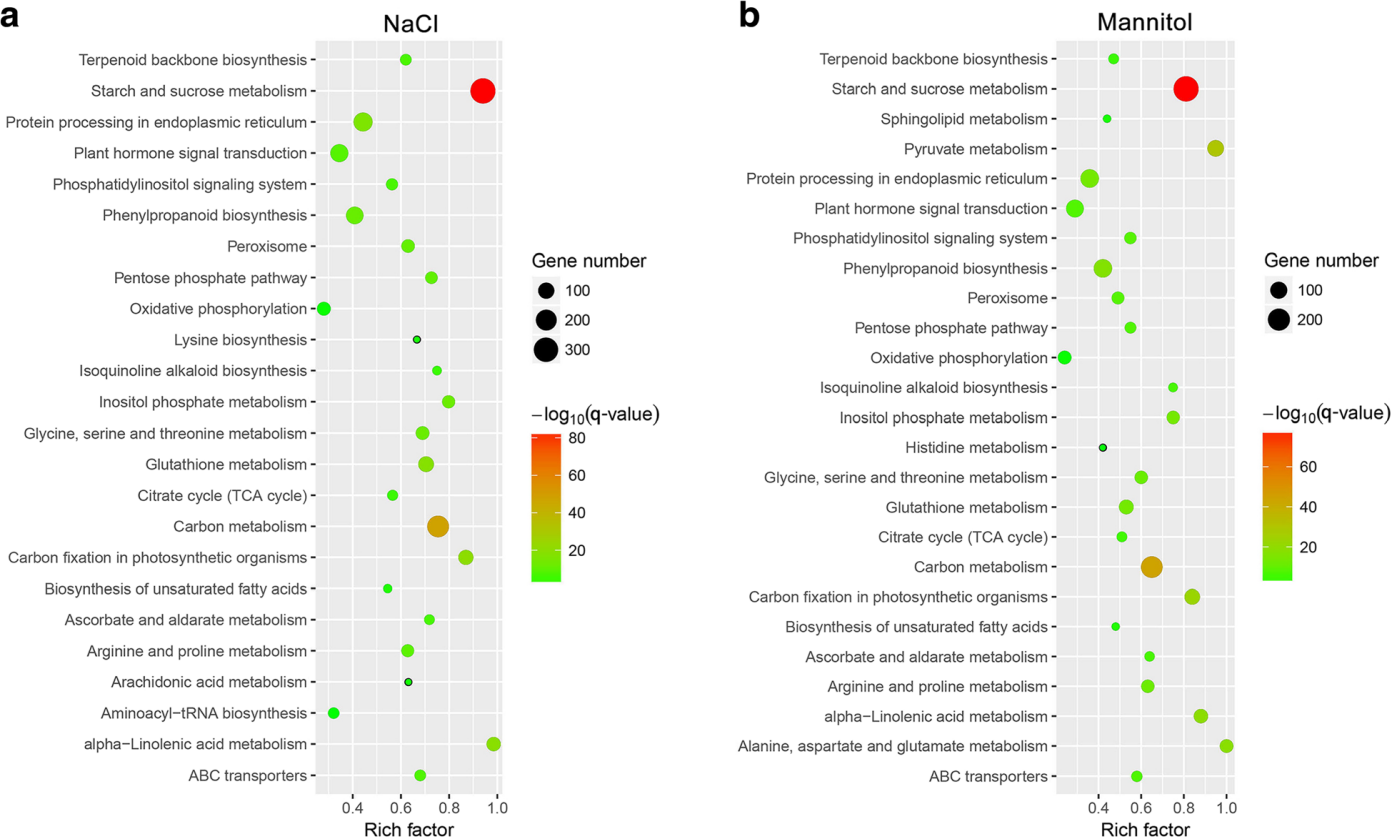
Scatterplot of enriched KEGG pathways for differentially expressed genes under NaCl and mannitol stress. The DEGs from all analyzed time points of NaCl (**a**) and mannitol (**b**). The rich factor is the ratio of the DEG number to the total gene number in a certain pathway. The size and colour of the dots represent the gene number and the range of the -log_10_ (*q*-value), respectively

KEGG pathway enrichment analysis were performed to understand the characteristics of the complex biological behaviour observed in the transcriptome profiles. Figure 7 presents the overall response pathways of alfalfa to NaCl and mannitol. Twenty-one common features involved in signal transduction, redox, and metabolic pathways were observed by pairwise comparisons of both stress responses, including “phosphatidylinositol signalling system”, “plant hormone signal transduction”, “ABC transporters”, “peroxisome”, “protein processing in endoplasmic reticulum”, “glutathione metabolism”, and “arginine and proline metabolism”. A few differences between the two stress responses were also observed. The DEGs under NaCl stress are involved mainly in biosynthesis, including three NaCl-specific pathways: “lysine biosynthesis”, “aminoacyl-tRNA biosynthesis”, and “arachidonic acid metabolism”. More metabolism-related DEGs were enriched after mannitol stress, including four mannitol-specific pathways: “pyruvate metabolism”, “alanine, aspartate and glutamate metabolism”, “sphingolipid metabolism”, and “histidine metabolism”.

## Discussion

### Sequence quality and annotation

The use of full-length libraries with long SMRT sequencing reads enabled the generation of full-length transcripts, which would greatly enhance the accuracy of genome annotation and transcriptome characterization, and would be beneficial for subsequent functional studies of important loci in plants [30]. In this study, for the first time, we analyzed the transcript profiles of alfalfa roots under continuous NaCl and mannitol treatment using the PacBio RS II platform. We identified a total of 52,787 full-length transcripts in the four libraries; the average length of the transcripts was 2551 bp, and some transcripts even reached 7.5 kb, which is much longer than the alfalfa transcriptome sequences lengths, such as 418 bp [23], 612 bp [31], 651 bp [32], 746 bp [33], and 803 bp [13], that have been obtained via previously reported NGS platforms. These high-quality full-length transcripts can efficiently facilitate the de novo transcriptome assembly of alfalfa. Of these full-length transcripts, more than 99% exhibited significant similarity (BLAST, *E*-value ≤10^− 5^) to genes in public databases (Additional file 2: Table S1), which is a higher percentage than that identified in other plants such as *Campeiostachys nutans* (78.01%) [34], *Vicia sativa* (66.10%) [35], and *Elymus sibiricus* (79.81%) [36]. The remaining unannotated transcripts may represent an alfalfa-specific gene pool.

We also investigated the gene expression of alfalfa roots under continuous NaCl and mannitol treatment using the BGISEQ-500 platform. A total of 51,896 genes were identified in the 33 sample libraries. Each library contained more than 40,000 genes (Additional file 1: Figure S3), suggesting that the expression of most genes in the alfalfa root tips were stable and that there were fewer genes with time-specific expression during abiotic stress. The expression profiles of ten selected genes as measured by qRT-PCR were consistent with the RNA-Seq data (Fig. 3), which demonstrated that our sequencing data were reliable and could be used to identify transcripts that are differentially regulated in response to abiotic stress.

### Characteristics analysis of the osmotic and ionic components of salinity stress

Hyperosmotic stress is one of major harmful effects that derives from salinity stress. By analyzing the response to mannitol, we can separate out the important osmotic stress component from salinity stress (NaCl). In this study, 5937 DEGs were identified to as being shared by both NaCl and mannitol stresses (Fig. 4d), implicated a substantial common regulatory system or significant cross-talk between the NaCl-induced and mannitol-induced osmotic response pathways exists. In addition, the GO categories “carbohydrate metabolic process”, “transmembrane transporter activity”, and “oxidoreductase activity”, and the KEGG pathways “phosphatidylinositol signalling system”, “plant hormone signal transduction”, “peroxisome”, and “starch and sucrose metabolism” were significantly co-enriched under both stresses (Figs. 6 and 7), findings that are consistent with previous observations showing an overlap of the responses in *Arabidopsis* [37], rice (*Oryza sativa*) [38], and maize (*Zea mays*) [39], and *Camellia sinensis* [40]. These results indicated that carbohydrate metabolic processes were accelerated and the antioxidant defence system was activated through many hormones (such as ABA and ethylene) and secondary messengers (such as ROS and Ca^2+^) in response to osmotic stress.

Salinity stress involves osmotic as well as ionic components. These two sensory modalities are evident in that some responses to NaCl remain distinct from responses to mannitol [41]. In this study, 2924 and 2079 DEGs were specifically regulated at least one time point during NaCl and mannitol stress, respectively (Fig. 4d), suggesting that distinct molecular mechanisms underlying the response to NaCl and mannitol exist. Moreover, the DEGs participating in the processes of “ion homeostasis”, “iron ion transport”, and “antiporter activity” were significantly present under NaCl stress, whereas these porcesses were absent under mannitol stress (Fig. 6b, f, g). Thus, we can conclude that ionic stress is exclusive for NaCl stress, and ion transporters may be activated and redirected to reestablish cellular ion homeostasis in salinity-treated alfalfa.

### Osmotic stress-related DEGs

A high salinity concentration in the soil solution causes hyperosmotic pressure on plant roots. In general, the osmotic stress signals are perceived by several receptors at the cell membrane, such as receptor-like protein kinases (RLKs), followed by the synthesis of secondary messengers [42]. The change in secondary messengers stimulates downstream signals mediated by combinations of protein phosphorylation cascades, such as CDPKs, CIPKs, and mitogen activated protein kinases (MAPKs), which are central regulators in signal transduction, connecting the perception of external stimuli to cellular responses [43]. In this study, we identified that a total of 59 and 59 DEGs as being signal receptors or transducers after exposure to NaCl and mannitol, respectively (Additional file 2: Table S5). Among these signal sensors, 31 *RLKs*, 5 *CDPKs*, 12 *CIPKs*, and 1 *MAPK* were shared between the two stresses. The majority of all the 31 *RLKs* were first up-regulated and then down-regulated, whereas the remaining genes, including 3 of the 5 *CDPKs*, 11 of the 12 *CIPKs*, and 1 *MAPK* were up-regulated after exposure to NaCl or mannitol for 24 h (Additional file 1: Figure S5a), indicating that signal perception and transduction may be highly activated during early osmotic stress stage.

The ABA signalling pathway is also activated during early stages of osmotic stress. In the presence of ABA, PYL receptors bind to ABA and prevent PP2C-mediated dephosphorylation of SnRK2, causing the activation of SnRK2 kinases. Active SnRK2s can phosphorylate downstream TFs and can activate the expression of ABA-dependent genes, thus eliciting ABA responses [44]. In this study, all of the key genes involved in the abovementioned ABA signalling were identified from the “plant hormone signal transduction” KEGG pathway (Fig. 7; Additional file 2: Table S6). A total of 4, 9, and 3 *PYLs*, *PP2Cs*, and *SnRK2s* was common to both stresses. The transcript abundances for the 4 *PYLs* were all inhibited during the 24-h NaCl or mannitol treatment, whereas the remaining genes, including 9 *PP2Cs* and 3 *SnRK2s* were uniformly upregulated after treatment with NaCl or mannitol for 24 h (Additional file 1: Figure S5b). These results are in agreement with what is known of the ABA regulation pathway [45], indicating that osmotic stress can activate the ABA signalling pathway and that it affected the PYLs, PP2Cs, and SnRK2s.

TFs, which are crucial components in osmotic stress-mediated signalling pathways, are generally phosphorylated by protein kinases and directly control the expression of specific sets of downstream stress-responsive genes [46]. Within the alfalfa transcriptome, at least 188 TFs in 32 TF families, such as the *GRAS*, *MYB*, *AP2-EREBP*, *NAC*, *WRKY*, and *bHLH* families, were identified as being DEGs under the two stresses (Fig. 5; Additional file 2: Table S4). These results are consistent with a previous report on alfalfa [22]; however, the number of TFs is higher than that previously reported (102 TFs), which may be because a more comprehensive time-course coverage for stress (from 1 to 24 h) was used in our study. The GRAS TF family has been implicated in various biological processes, such as root and meristem development, light signalling, and biotic stress and abiotic stress responses [47]. In this study, the largest class of TFs induced by NaCl (28) and mannitol (26) stress was the *GRAS* family. Of these *GRAS* family members, a total of 20 were common to both stresses, and the diverse expression of these DEGs suggested that alfalfa may reduce osmotic damage by regulating root and meristem development (Additional file 1: Figure S5c). The MYB family is another group of TFs that are involved in plants’ response to environmental stresses [48]. Genetic analysis showed that an overexpression of *OsMYB2* in rice not only resulted in an ABA-hypersensitive phenotype but also improved the salinity, cold, and dehydration tolerance of the transgenic plants [49]. In this transcriptome analysis, a total of 21 and 15 *MYBs* were identified for NaCl and mannitol, respectively. Of these *MYBs*, 14 were common to both stresses; and 10 of these were up-regulated, and the other 4 were down-regulated (Additional file 1: Figure S5c), thus indicating that MYB family has great biological importance in osmotic tolerance. Moreover, TFs such as AP2-EREBPs, NACs, WRKYs, and bHLHs are believed to participate in the abiotic stress responses and tolerances in many plant species [42]. These TFs showed both inducible and suppressed expression patterns after NaCl and mannitol stress (Additional file 1: Figure S5c), suggesting that sophisticated transcriptional regulation could participate in the adaptation of alfalfa to adverse environments.

**Fig. 8:**
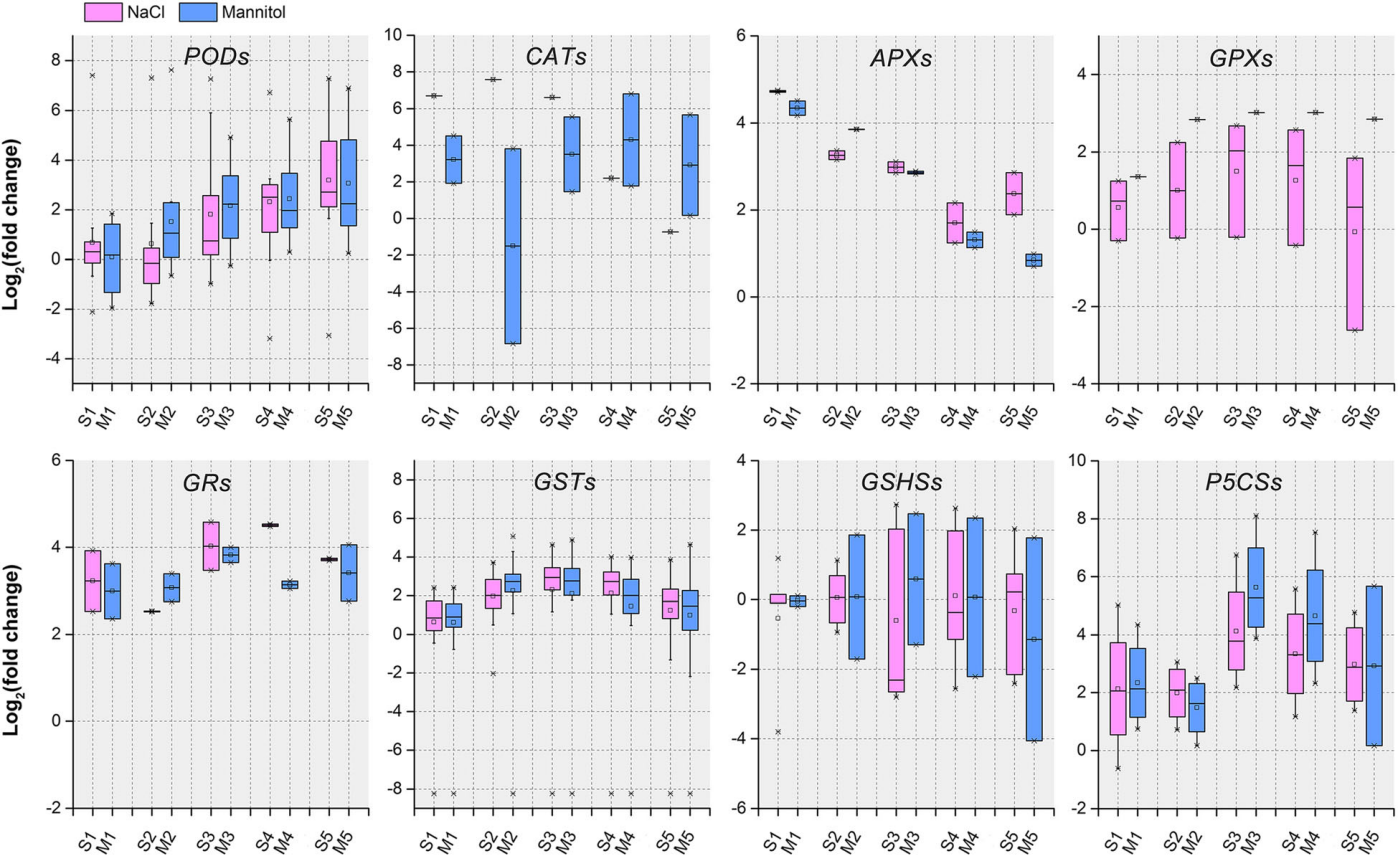
Boxplot indicating the expression changes of the antioxidant defense-related DEGs co-regulated by NaCl and mannitol stress. The boxes show the interquartile range (IQR) between 25% (Q1) and 75% (Q3) of the values; the open quadrates are average values; the thick horizontal black bars are the median values; the whiskers define the “fence” = [Q1, Q3] + 1.57 × IQR; and the crosses are outliers beyond the fence

Under osmotic stress, ROS as toxic products, which result in oxidative damage and cell death [50]. Plants sense the increased production of ROS using redox-sensitive TFs and other molecular sensors and activate different ROS defense/metabolic pathways for ROS scavenging, among which the antioxidant defense system prevails [46]. Our physiological work showed that both NaCl and mannitol stresses resulted in marked oxidative damage in alfalfa, meaning that the chlorophyll content decreased markedly, ion leakage and lipid peroxidation were significantly aggravated, and ROS levels accumulated markedly (Fig. 1). To alleviate oxidative damage, alfalfa significantly activated the antioxidant defence system using ROS detoxification of antioxidants (POD, CAT, and GSH) and osmotic adjustment substance (PRO) to maintain cellular ROS (mainly including H_2_O_2_) at relatively low levels (Fig. 2). Consistently, our transcriptome analysis also revealed that the antioxidant defense system was activated at the molecular level by NaCl and mannitol stress. As shown in Figs. 6 and 7, the GO category “oxidoreductase activity” and the KEGG pathways “peroxisome” and “glutathione metabolism”, and “arginine and proline metabolism” were significantly co-enriched after expose to NaCl and mannitol. Additionally, the majority of antioxidative enzymes-related DEGs, such as *PODs*, *CATs*, *APX*s, *GPXs*, and *GRs*, non-enzymatic antioxidants-related DEGs, such as *GSTs* and *GSHSs*, and PRO synthetases-related DEGs, such as *P5CSs*, (Fig. 8; Additional file 1: Figure S5d; Additional file 2: Table S7), were significantly modulated by both stresses, which is consistent with previous reports on alfalfa under salinity stress [23]. These results strongly suggested an important role for these DEGs in response to osmotic stress and thus protected alfalfa from ROS damage.

### Ionic stress-related DEGs

High salinity levels can also cause ionic stress on plant roots. Ionic stress (mainly Na^+^) triggers an increase in cytosolic Ca^2+^, and thereafter, Ca^2+^-binding proteins further activate downstream signalling pathways. CBL-CIPKs signalling modules are one of central calcium signalling mechanisms which regulate plant responding to salinity stress, as well as drought, heat, cold, and low nutrient availability [51].

**Fig. 9:**
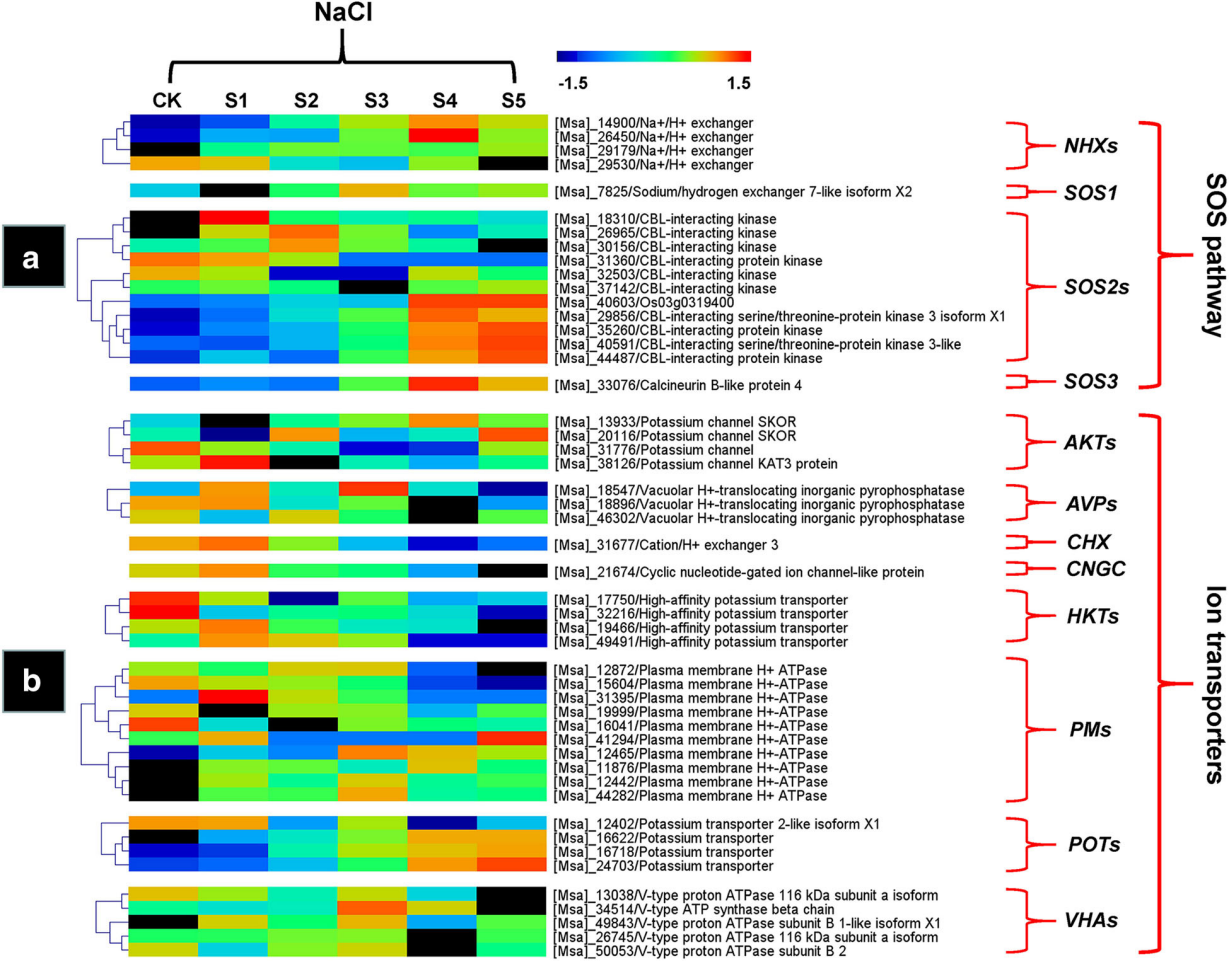
Heatmap plot of the expression levels of the ionic stress-related DEGs under NaCl stress. The gene expression is based on the z-scores of log_2_ (FPKM) value. **a**. SOS pathway-related DEGs. **b**. Ion transporters-related DEGs. The red and blue colors indicate high and low expression levels, respectively

The SOS pathway is an example of a CBL-CIPK signalling pathway, was first established in plants, and is specific for salinity [52]. In this pathway, the Ca^2+^ sensor CBL4/SOS3 activates the serine/threonine protein kinase CIPK24/SOS2, which positively regulates the plasma membrane-localized Na^+^/H^+^ antiporter SOS1, which is also defined as NHX7 [7]. Genetic evidence indicates that mutations in SOS1, SOS2, or SOS3 all reduce the Na^+^/H^+^ exchange activity, and a constitutively active SOS2 enhances Na^+^/H^+^ exchange activity in a SOS1-dependent and SOS3-independent manner [53]. Moreover, SOS1 was reported to export Na^+^ out of the cell, whereas most of the other NHX members are essential for Na^+^ detoxification via sequestration of Na^+^ within the vacuole [41]. Both types of exchangers are required for the intracellular and intercellular ion balance mechanism. In the present NaCl-related transcriptome analysis, the GO categories “iron ion transport”, “ion homeostasis”, and “antiporter activity” were significantly enriched after NaCl stress (Fig. 6b, f, g), and totals of 4, 1, 11, and 1 DEGs that showed similarity to *NHXs*, *SOS1s*, *SOS2s*, and *SOS3s*, respectively (Fig. 9a; Additional file 2: Table S8). The up-regulation of nearly all of these four types of DEGs suggested both the intracellular and intercellular Na^+^ detoxification mechanism were activated in alfalfa roots. However, a previous study of salinity-treated alfalfa leaf transcriptome reported that some vacuolar membrane NHXs, rather than plasma membrane SOS1, were induced to very high levels in response to salinity stress [23], indicating alfalfa mainly compartmentalize excessive cytosolic Na^+^ into vacuoles via the corresponding vacuolar NHXs in leaves. This finding thus favors a scenario in which a different Na^+^ detoxification mechanism exists in alfalfa roots and leaves.

The CBL-CIPK modules can also interact with and regulate the activity of a number of ion transporters, particularly Na^+^/K^+^ transporters, which are essential for ion homeostasis [54]. In many cases, these ion transporters interact synergistically or antagonistically with the SOS signalling network, thereby maintaining the “balance” of these cations in plants under salinity stress condition. In this study, the expressions of many ion transporters, including 4 *AKT/KATs*, 3 *AVPs*, 1 *CHX*, 1 *CNGC*, 4 *HKTs*, 10 *PMs*, 4 *POTs*, and 5 *VHAs*, were affected by NaCl (Fig. 9b; Additional file 2: Table S9), indicating that these DEGs may directly or indirectly contribute to alfalfa cellular Na^+^ detoxification mechanisms.

## Conclusions

**Fig. 10:**
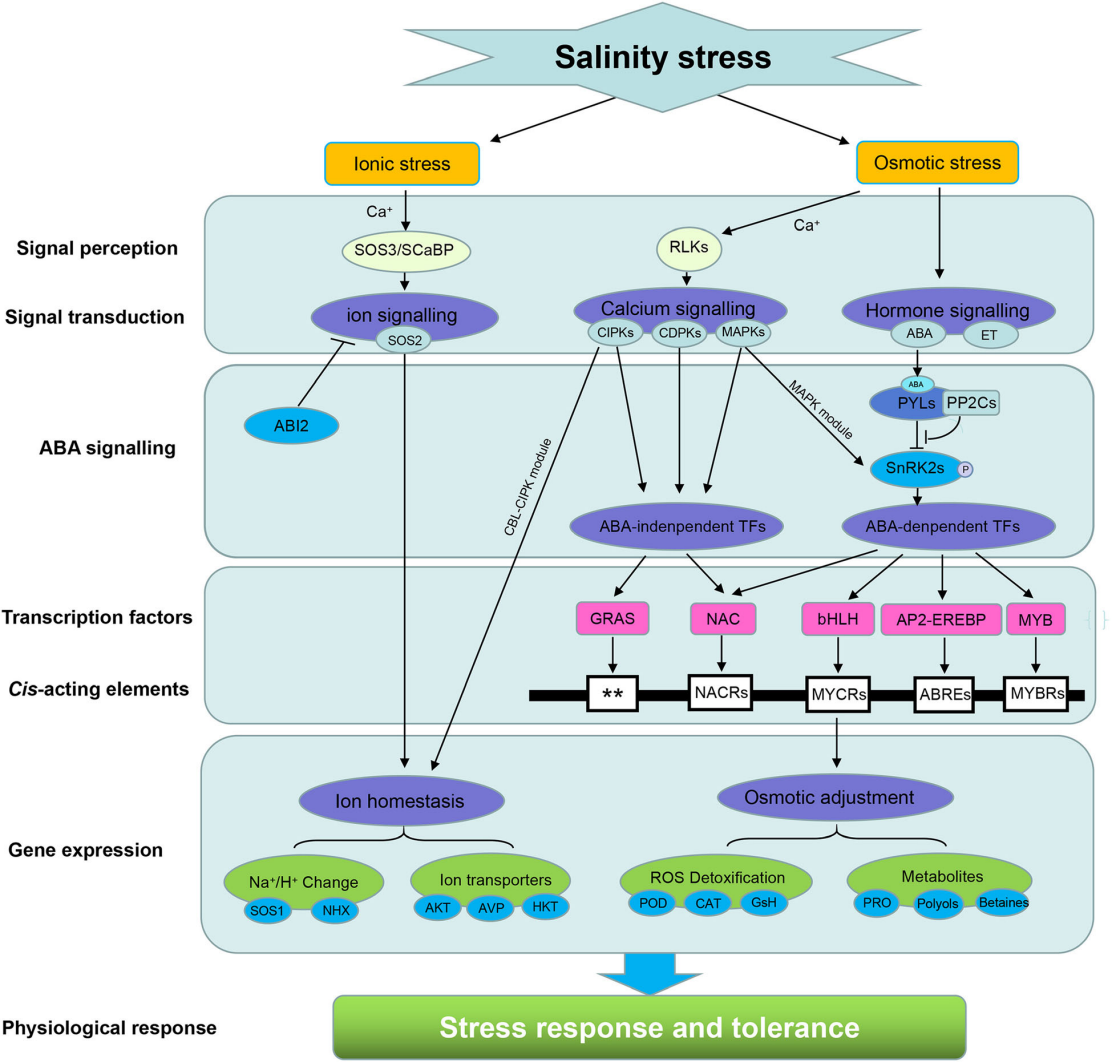
Models describing the signalling pathways involved in the acquisition of salinity tolerance in alfalfa. The broken arrows indicate speculative regulation, and the solid arrows indicate activation, whereas lines ending with a bar show negative regulation. The two stars (**) indicate unknown cis-elements

In this study, we presented the first full-length transcript sequencing and comprehensive transcriptome analysis for alfalfa root tips under a prolonged time-course for NaCl and mannitol stress. These sequences were assembled into 52,787 all-full-length transcripts, with an average length of 2551 bp. Next, a total of 8861 NaCl-regulated and 8016 mannitol-regulated DEGs were identified and analyzed for their potential role in the response to abiotic stress using clustering, GO and KEGG enrichment analyses. These DEGs overlapped or diverged in the cascades of molecular networks involved in signal perception, signal transduction, transcriptional regulation, and antioxidative defense (Fig. 10). The CDPK, MAPK, CIPK, and PYL-PP2C-SnRK2 core pathways were shown to be involved in osmotic stress, while the SOS pathway was activated by ionic stress. The antioxidant defense system plays important roles in alfalfa response to salinity stress via analyzing the expression pattern of ROS-detoxification-related genes and their physiological changes. These findings provide valuable information for further investigations of key genes and molecular mechanisms involved in the adaptation of alfalfa to salinity stress.

## Methods

### Plant materials and growth conditions

Alfalfa seeds of Zhongmu No. 1 was kindly provided by Prof. Qingchuan Yang (Institute of Animal Sciences, Chinese Academy of Agricultural Sciences, Beijing, China). Zhongmu No. 1, which has been bred on saline–alkaline land, has been widely cultivated as a salinity-tolerant cultivar and should thus be an ideal material for understanding the physiological and transcriptional shifts under salinity stress [55]. Seeds of Zhongmu No. 1 were surface sterilized and then placed on sterilized filters that were moistened with 5 ml of distilled water in 12 cm × 12 cm square Petri dishes at 22 °C for 5 days. To ensure that the roots of the seedlings grew in a straight manner, the Petri dishes were turned upside down. Thereafter, 48 seedlings with uniform taproot lengths were separately sown in 96-well plates supported by a plastic container and then hydroponically grown in half-strength Murashige and Skoog (1/2 MS) nutrient solution at a pH of 5.8. The containers were subsequently moved to a controlled-environment chamber whose conditions included a 22 °C temperature, 80% relative humidity, 180 μmol m^− 2^ s^− 1^ photosynthetically active radiation and a 16 h light/8 h dark photoperiod for 1 week. During seedling growth, the nutrient solution was changed every 2 days.

### NaCl and mannitol treatment

To optimize NaCl and mannitol concentrations, seven-day-old seedlings were exposed to the 1/2 MS nutrient solution containing various concentrations of NaCl (200, 250, 300, and 350 mM) or mannitol (300, 400, 500, and 600 mM) for 7 days. The control seedling were grown in normal 1/2 MS nutrient solution. Fresh weight and survival rate of the seedlings were measured. Three independent experiments were performed.

Twelve-day-old seedlings were separated into three groups: (1) control, (2) NaCl, (3) mannitol groups. The seedlings were then treated with 1/2 MS nutrient solution that contained 250 mM NaCl or 400 mM mannitol for 5 exposure time points (1, 3, 6, 12, and 24 h), and the control seedlings were grown in normal 1/2 MS nutrient solution; all 11 treatments reached the final sampling period at the same time (Additional file 2: Table S10). Three biological replicates were included for each treatment time point, including the control group. To reduce circadian rhythm effects, the seedlings were continuously exposed to light throughout all treatment time points. After treatment, the leaves (trifoliate leaves) and root tips (approximately 1.5 cm in length; a pool of 20 different root tips) were selected for physiological analysis. Moreover, other root tips were selected for sequencing analysis; those root tips were immediately frozen in liquid nitrogen and then stored at − 80 °C.

### Determination of physiological characteristics

All 33 samples were immediately assessed via eight physiological indexes. For the leaves, the chlorophyll content was measured by ultraviolet absorption methods, and the electrolyte leakage was determined with a DDS-309+ conductivity meter (Chengdu century Ark Technology Co.Ltd., Chengdu, Sichuan, China). For the roots, the contents of MDA, H_2_O_2_, GSH, and PRO, and the enzyme activities of POD and CAT were determined using Comin Biochemical Test Kits (MDA-2-Y, H_2_O_2_–2-Y, GSH-2-W, PRO-2-Y, POD-2-Y, and CAT-2-Y, respectively; Cominbio, Suzhou, Jiangsu, China; http://www.cominbio.com/) in accordance with the manufacturer’s instructions.

### Iso-Seq library preparation, sequencing, assembly, and annotation

The RNA extractions as well as the quality and quantity measurements of all 33 alfalfa samples were performed as previously described [32]. Equal amounts of total RNA with 2.2 ≥ A260/A280 ≥ 1.9, a 28S/18S ratio ≥ 1.0, and an RNA integrity number ≥ 8.0 from all 33 alfalfa samples were equally pooled together to prepare the Iso-Seq library as described by Pacific Biosciences with the following modifications. For cDNA conversion, 4 μg of total RNA was used to synthesize first-strand cDNA via a Clontech SMARTer PCR cDNA Synthesis Kit (Clontech, Mountain View, CA, USA), followed by large-scale PCR to synthesize second-strand cDNA. Four barcoded SMRTBell libraries (1–2 kb, 2–3 kb, 3–6 kb, and 5–10 kb) were size-selected with a BluePippin™ system (Sage Science, Beverly, MA, USA) to remove trace amounts of small inserts. The cDNA products were then subjected to construction of SMRTBell template libraries by annealing a sequencing primer [component of SMRTBell Template Prep Kit 1.0 (PacBio, Menlo Park, CA, USA)] and binding polymerase to the primer annealed template [56, 57]. Finally, a total of 12 SMRT cells were sequenced on the PacBio RSII platform using P6-C4 chemistry with 4–6 h movies.

The raw data from the four libraries were processed according to the PacBio transcriptome analysis procedure using SMRT Analysis (version 2.3.0) software (PacBio, USA). In this pipeline, the raw polymerase reads were first partitioned into sub-reads, which consisted of the inserted cDNA sequence. Reads of inserts (ROIs) were generated using the default number of full passes for the sub-reads. Then, the ROIs were classified into full-length if the 5′ and 3′ sequencing primers as well as the poly(A) tail were simultaneously observed. In the cluster panel, the options of “Predict *de novo* Consensus Isoforms using the ICE Algorithm” and “Call Quiver to Polish Consensus Isoforms” were applied to obtain *high QV*, full-length, and polished consensus transcripts. Finally, the *high QV* consensus transcripts of multiple libraries were merged together, and the redundancy was removed to obtain the final set of full-length transcripts, which represents a comprehensive reference transcriptome database referred to as “MSA”.

For further analysis, all full-length transcripts were annotated into seven public databases, including the NCBI Nr, NCBI Nt, SwissProt, InterPro, COG, GO, and KEGG databases. The CDSs of the transcripts were also predicted by the protein databases via BLASTx (*E*-value ≤10^− 5^) in the priority order of NR, SwissProt, KEGG, and COG; the transcripts that could not be aligned to any abovementioned databases were predicted by ESTScan. The shortest CDS was at least 100 bp.

### BGISEQ-500 RNA-Seq

The total RNA from all 33 alfalfa samples was then separately prepared for BGISEQ-500 RNA-Seq. In brief, the total RNA was purified to enrich poly(A) mRNA with magnetic oligo (dT) beads. The target RNA was fragmented and then used for dscDNA library construction by random hexamer (N6) primers. The ends of the dscDNA were repaired with phosphate at the 5′ end and sticky ‘A’ at the 3′ end, after which the dscDNA strands were ligated with adapters that had a sticky ‘T’ at the 3′ end. Two specific primers were used to amplify the ligation product. Finally, the PCR product was denatured by heat, and the single-strand DNA was cyclized by splint oligo and DNA ligase. Thirty-three cDNA libraries were constructed and sequenced on a BGISEQ-500 RS platform at BGI Shenzhen.

After quality control checks, clean reads were separated from the raw data by removing adaptor sequences, reads with more than 10% of unknown bases, and low-quality reads. The high-quality clean reads were then mapped to the “MSA” reference full-length transcriptome database via Bowtie2 software. The gene expression level was quantified by the RSEM software package [58] and was normalized by the FPKM method [59].

### qRT-PCR analysis

The total RNA of all 33 alfalfa samples used for the transcriptome analysis was also used to make cDNAs for qRT-PCR validation. Briefly, a FastQuant RT Kit (with gDNase) (Tiangen Biotech, Beijing, China) was used in accordance with the manufacturer’s instructions to generate cDNA from 1 μg of total RNA from each sample. qRT-PCR was performed using 2xSG Fast qPCR Master Mix (Sangon Biotech, Shanghai, China) on a 7500 Fast Real-time PCR system (Applied Biosystems, Foster City, CA, USA). *MsUBQ* was selected as an internal control gene, owing to its relatively stable expression in alfalfa transcription profiles [60]. Gene-specific primers for qRT-PCR were designed via DNAMAN software (Lynnon BioSoft, Vandreuil, Quebec, Canada) and are shown in Additional file 2: Table S11. The qRT-PCR analysis of each sample was performed in triplicate. The expression levels of each gene were normalized to those of *MsUBQ*, and the relative gene expression levels were calculated according to the 2^-∆∆Ct^ method.

### DEG analysis

Based on the average FPKM values in each treatment, differential expression between the treatment group and the control group was assessed via the NOISeq program [61]. Both the absolute values of the log_2_(fold change) ≥ 2 and the divergence probability ≥0.8 were used as thresholds to identify significant DEGs. The cluster analysis and expression pattern assessment for DEGs were performed by MEV 4.9 software (https://sourceforge.net/projects/mev-tm4/files/mev-tm4/) via the hierarchical clustering and the K-means clustering methods, respectively. For functional annotation analyses, GO and KEGG pathway enrichment analyses for DEGs were conducted via agriGO 2.0 (http://systemsbiology.cau.edu.cn/agriGOv2/) and KOBAS 3.0 (http://kobas.cbi.pku.edu.cn/), respectively. TFs were predicted and classified into different families using the PlantTFDB (http://plntfdb.bio.uni-potsdam.de/v3.0/) and the cluster analysis for TFs was conducted using the Self-Organizing Tree Algorithm in the MEV4.9 software.

## Additional files

Additional file 1: **Figure S1.** Investigation of the characteristics of NaCl and mannitol stress resistance in alfalfa seedlings. **Figure S2.** Iso-Seq sequencing, assembly, and annotation of alfalfa. **Figure S3.** The quality of the BGISEQ-500 RNA-Seq data. **Figure S4.** An overview of continuous dynamic changes in DEG expression levels. **Figure S5.** Heatmap plot of the expression levels of the osmotic stress-related DEGs co-regulated by NaCl and mannitol. (PDF 875 kb)

Additional file 2: **Table S1.** BLAST analysis of the full-length transcripts against public databases. **Table S2.** Summary of the BGISEQ-500 RNA-Seq data analysis. **Table S3.** DEGs commonly or specifically regulated at all analyzed time points under NaCl and mannitol stress. **Table S4.** TFs that are differentially expressed due to NaCl and mannitol. **Table S5.** DEGs involved in signal sensors under NaCl and mannitol stress. **Table S6.** DEGs involved in the ABA signal transduction pathway under NaCl and mannitol stress. **Table S7.** DEGs involved in antioxidative defense under NaCl and mannitol stress. **Table S8.** DEGs involved in the SOS pathway under NaCl stress. **Table S9.** DEGs encoding ion transporters under NaCl stress. **Table S10.** Experimental design of alfalfa seedlings. **Table S11.** Primers used for qRT-PCR analysis. (XLSX 140 kb)

## Abbreviations

1/2 MS: Half-strength murashige and skoog
ABA: Abscisic acid
AKT/KAT: K^+^ inward rectifying channel
APX: Ascorbate peroxidase
AVPq: Vacuolar H^+^-translocating inorganic pyrophosphatase
CAT: Catalase
CDPK: Calcium-dependent protein kinase
CHX: Cation-proton exchanger
CIPK: CBL-interacting protein kinase
CNGC: Cyclic nucleotide-gated ion channel
COG: Clusters of Orthologous Groups of proteins
COR15A: Cold-regulated 15A
DEG: Differentially expressed gene
FPKM: Fragments per kilobase per million fragments mapped
GO: Gene ontology
GPX: Glutathione peroxidase
GR: Glutathione reductase
GSH: Glutathione
GSHS: Glutathione synthase
GST: Glutathione S-transferase
HKT: High-affinity K^+^ transporter
Iso-Seq: Isoform sequencing
KEGG: Kyoto encyclopedia of genes and genomes
MAPK: Mitogen activated protein kinase
MDA: Malonaldehyde
NCBI: National Center for Biotechnology Information
NGS: Next-generation sequencing
NHX: Na^+^/H^+^ exchanger
Nr: Non-redundant protein sequence
Nt: Non-redundant nucleotide sequence
P5CS: Δ1-pyrroline-5-carboxylate synthetase
PM: Plasma membrane H^+^-ATPase
POD: Peroxidase
POT: Potassium transporter
PP2C: Type 2C protein phosphatase
PRO: Proline
PYL: Pyrabactin resistance 1-like family
qRT-PCR: Quantitative real-time PCR
RLK: Receptor-like protein kinase
ROI: Reads of insert
ROS: Reactive oxygen specie
SMRT: Single molecule real-time
SnRK2: Sucrose nonfermenting1-related protein kinase 2
SOS: Salt overly sensitive
TF: Transcription factor
VHA: V-type H^+^-transporting ATPase
