# Supplementary material for: Full-length transcript sequencing and comparative transcriptomic analysis to evaluate the contribution of osmotic and ionic stress components towards salinity tolerance in the roots of cultivated alfalfa (Medicago sativa L.)
Source: BMC Plant Biol. 2019 Jan 21;19:32. doi: 10.1186/s12870-019-1630-4 (PMC6341612; doi:10.1186/s12870-019-1630-4)
Supplement: Supplementary file 1 — Figure S1. Investigation of the characteristics of NaCl and mannitol stress resistance in alfalfa seedlings. Figure S2. Iso-Seq sequencing, assembly, and annotation of alfalfa. Figure S3. The quality of the BGISEQ-500 RNA-Seq data. Figure S4. An overview of continuous dynamic changes in DEG expression levels. Figure S5. Heatmap plot of the expression levels of the osmotic stress-related DEGs co-regulated by NaCl and mannitol. (PDF 875 kb) [file 12870_2019_1630_MOESM1_ESM.pdf]

Full-length transcript sequencing and comparative transcriptomic analysis to evaluate the contribution of osmotic and ionic stress components towards salinity tolerance in the roots of cultivated alfalfa (*Medicago sativa* L.)

Dong Luo, Qiang Zhou, Yuguo Wu, Xutian Chai, Wenxian Liu, Yanrong Wang, Qingchuan Yang, Zengyu Wang, and Zhipeng Liu

Additional file 1: This PDF contains all of the additional material (Figures S1–S5) associated with the manuscript. Figure numbers and titles are listed below:

Figure S1. Investigation of the characteristics of NaCl and mannitol stress resistance in alfalfa seedlings.

Figure S2. Iso-Seq sequencing, assembly, and annotation of alfalfa.

Figure S3. The quality of the BGISEQ-500 RNA-Seq data.

Figure S4. An overview of continuous dynamic changes in DEG expression levels.

Figure S5. Heatmap plot of the expression levels of the osmotic stress-related DEGs co-regulated by NaCl and mannitol.

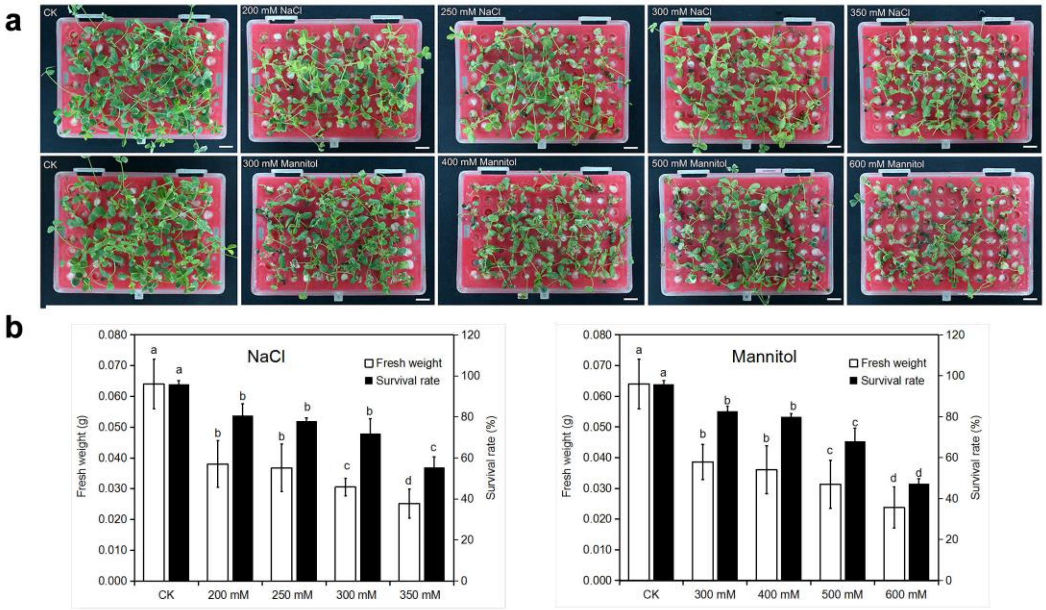

Figure S1. Investigation of the characteristics of NaCl and mannitol stress resistance in alfalfa seedlings. a Alfalfa phenotypes 7 days after the application of various concentrations of NaCl (200, 250, 300, and 350 mM) or mannitol (300, 400, 500, and 600 mM). **b** The fresh weight and survival rate of alfalfa under various concentrations of NaCl or mannitol.

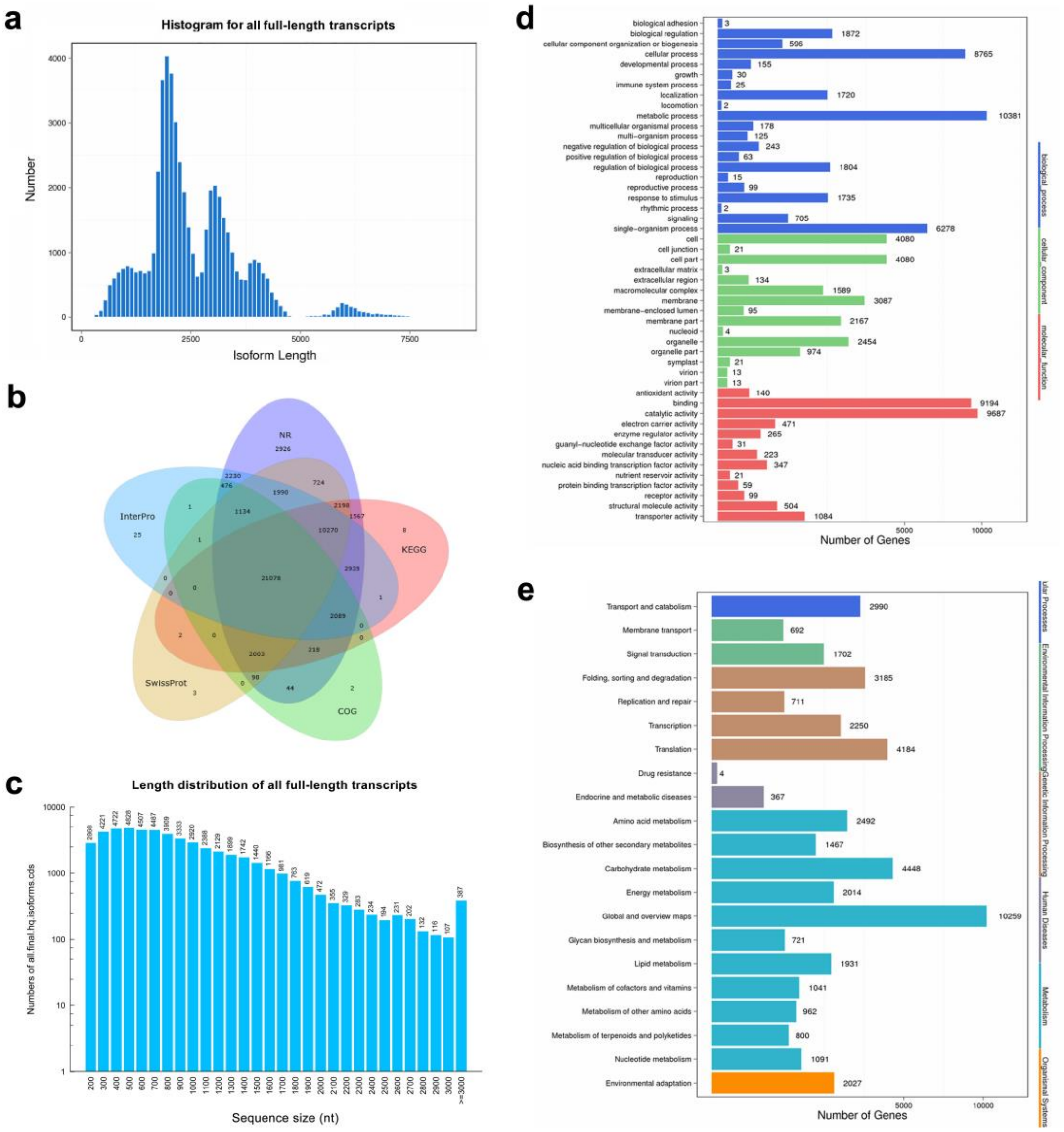

Figure S2. Iso-Seq sequencing, assembly, and annotation of alfalfa. a Length distribution of the assembled full-length transcripts. **b** Venn diagram of the full-length transcripts against five public libraries. **c** Length distribution of the CDS of the assembled full-length transcripts. **d** GO classification of the assembled full-length transcripts. **e** KEGG annotation of the assembled full-length transcripts.

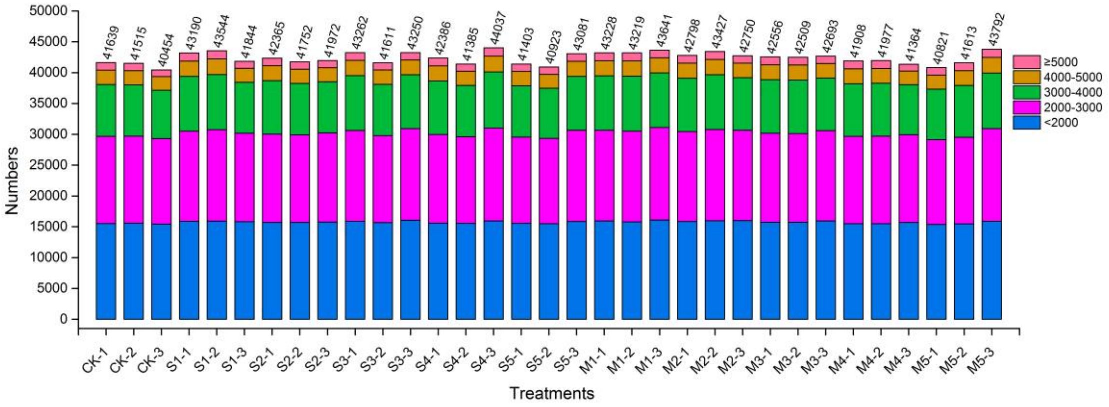

Figure S3. The quality of the BGISEQ-500 RNA-Seq data. CK represent control; S1, S2, S3, S4, and S5 represent 1, 3, 6, 12, and 24 h after NaCl treatment; M1, M2, M3, M4, and M5 represent 1, 3, 6, 12, and 24 h after mannitol treatment. Three biological replicates for each time points, including the control group.

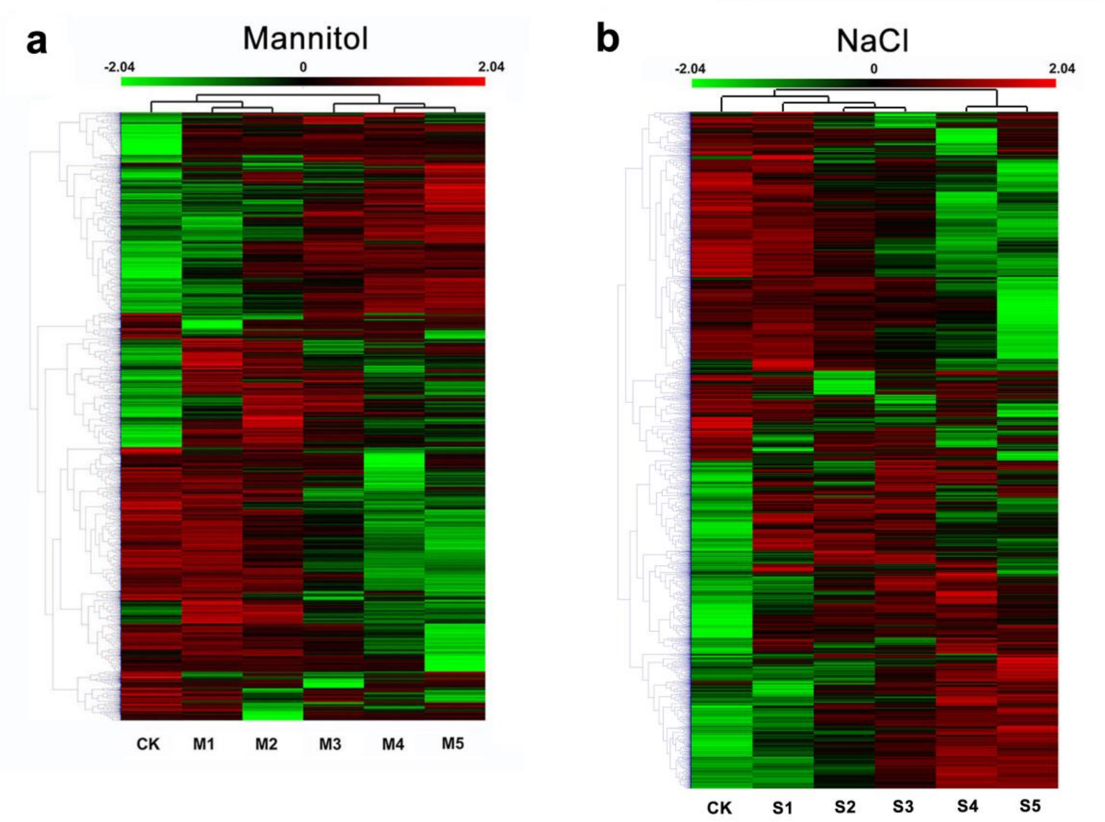

Figure S4. An overview of continuous dynamic changes in DEG expression levels. **a** NaCl-regulated DEGs. **b** Mannitol-regulated DEGs. Heat map showing all DEGs via MEV 4.9 software with the hierarchical clustering method. The gene expression is based on the z-scores of $\log_2$ (FPKM) value. The red and green colours indicate high and low expression levels, respectively.

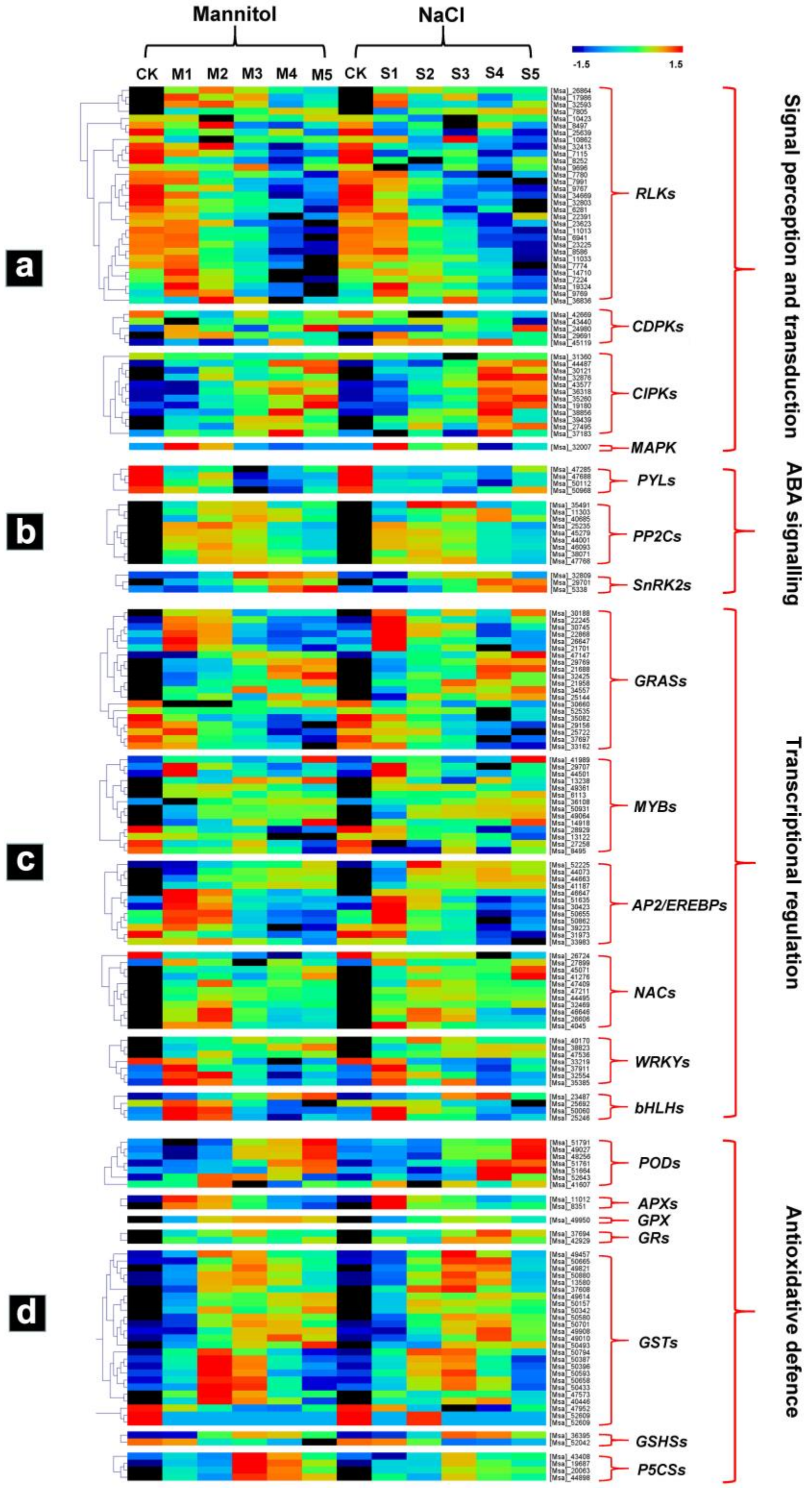

Figure S5. Heatmap plot of the expression levels of the osmotic stress-related DEGs co-regulated by NaCl and mannitol. a Signal sensors-related DEGs. b ABA signaling-related DEGs. **c** Transcriptional regulation-related DEGs. **d** Antioxidative defence-related DEGs. The gene expression is based on the z-scores of $\log_2$ (FPKM) value. The red and blue colors indicate high and low expression levels, respectively.
